# Striatin plays a major role in angiotensin II-induced cardiomyocyte and cardiac hypertrophy in mice *in vivo*

**DOI:** 10.1042/CS20240496

**Published:** 2024-05-22

**Authors:** Joshua J. Cull, Susanna T.E. Cooper, Hajed O. Alharbi, Sonia P. Chothani, Owen J.L. Rackham, Daniel N. Meijles, Philip R. Dash, Risto Kerkelä, Neil Ruparelia, Peter H. Sugden, Angela Clerk

**Affiliations:** 1 School of Biological Sciences, University of Reading, Reading, U.K.; 2 Molecular and Clinical Sciences Institute, St. George’s University of London, London, U.K.; 3 Program in Cardiovascular and Metabolic Disorders, Duke-National University of Singapore Medical School, Singapore; 4 School of Biological Sciences, University of Southampton, Southampton, U.K.; 5 Research Unit of Biomedicine and Internal Medicine, Medical Research Centre Oulu (Oulu University Hospital) and Biocenter Oulu, University of Oulu, Oulu, Finland; 6 Department of Cardiology, Royal Berkshire Hospital, Reading, U.K.

**Keywords:** Cardiac hypertrophy, Heart failure, hypertension, Protein kinase, Protein phosphatase 2A

## Abstract

The three striatins (STRN, STRN3, STRN4) form the core of ***STR***iatin-***I***nteracting ***P***hosphatase and ***K***inase (STRIPAK) complexes. These place protein phosphatase 2A (PP2A) in proximity to protein kinases thereby restraining kinase activity and regulating key cellular processes. Our aim was to establish if striatins play a significant role in cardiac remodelling associated with cardiac hypertrophy and heart failure. All striatins were expressed in control human hearts, with up-regulation of STRN and STRN3 in failing hearts. We used mice with global heterozygote gene deletion to assess the roles of STRN and STRN3 in cardiac remodelling induced by angiotensin II (AngII; 7 days). Using echocardiography, we detected no differences in baseline cardiac function or dimensions in STRN^+/−^ or STRN3^+/−^ male mice (8 weeks) compared with wild-type littermates. Heterozygous gene deletion did not affect cardiac function in mice treated with AngII, but the increase in left ventricle mass induced by AngII was inhibited in STRN^+/−^ (but not STRN3^+/−^) mice. Histological staining indicated that cardiomyocyte hypertrophy was inhibited. To assess the role of STRN in cardiomyocytes, we converted the STRN knockout line for inducible cardiomyocyte-specific gene deletion. There was no effect of cardiomyocyte STRN knockout on cardiac function or dimensions, but the increase in left ventricle mass induced by AngII was inhibited. This resulted from inhibition of cardiomyocyte hypertrophy and cardiac fibrosis. The data indicate that cardiomyocyte striatin is required for early remodelling of the heart by AngII and identify the striatin-based STRIPAK system as a signalling paradigm in the development of pathological cardiac hypertrophy.

## Introduction

The development of heart failure is associated with significant morbidity and a poor prognosis despite optimal medical therapy. It affects millions of people worldwide [1]. A leading cause of heart failure is hypertension that affects ∼30% of all adults [1,2]. Whilst the identification of patients suffering from hypertension and their management has significantly improved in recent years, a number of patients present acutely with end-organ damage or progress to complications of hypertension in spite of maximal therapy. Broader therapeutic options for individual patients to manage hypertensive heart disease are clearly needed, but this requires greater understanding of the underlying mechanisms. Striatin (STRN) is associated with salt-dependent hypertension in mice. Thus, mice that are heterozygotic for STRN gene deletion have a similar blood pressure profile to wild-type littermates when provided with a low salt diet, but have a greater increase in blood pressure with higher dietary salt [3]. This probably results from effects in endothelial cells and enhanced vasoconstriction [4]. These mice also have increased renal damage in response to aldosterone [5]. Further evidence for a role for STRN in heart failure comes from boxer dogs with arrhythmogenic right ventricular cardiomyopathy (ARVC) and heart failure, resulting from an 8 bp deletion in the 3′UTR of STRN and reduced STRN expression [6,7]. In humans, SNPs in the STRN gene are linked to blood pressure regulation [3,8,9], PR/QRS interval [10,11], hypertrophic cardiomyopathy [12] and heart failure [13]. SNPs in a second isoform, striatin 3 (STRN3, also known as SG2NA) are also linked to hypertension [14]. STRN, STRN3 and the third isoform, STRN4, are all expressed in the heart. Understanding the roles of these proteins may increase therapeutic options in patients with hypertension to both reduce the risk and to manage the development of heart failure complications.

The heart mainly contains contractile cardiomyocytes, endothelial cells in the capillary network and fibroblasts producing extracellular matrix [15]. Since adult mammalian cardiomyocytes are terminally-differentiated, they respond to increased cardiac workload (e.g., resulting from hypertension) with hypertrophic growth to increase contractile function [16]. The increase in cell size is associated with increased contractile apparatus and changes in gene expression (e.g., up-regulation of *Myh7*, *Nppa* and *Nppb* mRNAs). Cardiomyocytes may undergo necrosis or programmed cell death in situations of extreme or prolonged stress, compromising cardiac function and leading to heart failure [17]. Loss of endothelial cells and capillary rarefaction is another feature of developing heart failure [18], along with increased fibrosis in the myocardium (interstitial fibrosis) that compromises cardiac function by increasing ventricular wall stiffness, and around the arterioles (perivascular fibrosis) [19,20]. Fibrosis is associated with increased numbers of myofibroblasts which may derive from activation of resident fibroblasts or other cardiac cells (e.g., endothelial cells may undergo endothelial to mesenchymal transition and increase numbers of myofibroblasts) [21,22].

Cellular changes associated with cardiac remodelling are regulated by protein phosphorylation/dephosphorylation. Whilst much is known about protein kinase signalling cascades, specific roles of phosphatases and dephosphorylation are less well understood. The Ser-/Thr- phosphatase PP2A is highly abundant and ubiquitously expressed [23]. It is formed of one of two catalytic subunits (PP2A_C_), one of two regulatory subunits (PP2A_A_) and one of many targeting ‘B’ subunits that direct PP2A to its substrates. Striatins form a class of B subunits (B’’’) for PP2A, but also interact with protein kinases, particularly those of the Germinal Centre Kinase (GCK) family [24]. Because of this, striatin-based complexes have been termed ***STR***iatin-***I***nteracting ***P***hosphatase ***A***nd ***K***inase (STRIPAK) complexes [25,26]. This system places GCKs in close proximity to PP2A which maintains the kinase in a dephosphorylated (inactive) state. Inhibition of PP2A or removal of the phosphatase or kinase results in kinase activation, most probably through autophosphorylation. Striatins have an N-terminal domain that binds caveolins, potentially directing them to the plasma membrane and a Ca^2+^/calmodulin-binding domain. A coiled-coil domain and C-terminal WD repeats facilitate binding of other proteins. Interactome studies have identified many proteins in STRIPAK complexes, some of which probably direct different complexes to different subcellular targets and/or subdomains [27–29]. Striatins, GCKs and STRIPAKs regulate a diverse array of cellular processes including cell survival/proliferation/migration, key features of cardiac remodelling. As described above, STRN is linked to various forms of heart failure and conductance irregularities. Consistent with the latter, STRN is of significant importance in composite junctions between cells, and immunostaining experiments place STRN at intercalated discs between cardiomyocytes, potentially regulating ion fluxes between cells [6,30].

Our hypothesis is that STRIPAKs play a significant role in cardiac remodelling associated with developing heart failure. Here, we show that *STRN*, *STRN3* and *STRN4* are all dysregulated in human failing hearts compared with normal controls, with up-regulation of STRN and STRN3. Our data in mouse models with heterozygote knockout of STRN and STRN3 identified STRN, but not STRN3, as a potential mediator of the early phase of cardiac remodelling (i.e., prior to heart failure development and cardiac dysfunction) induced by developing hypertension in mice resulting from angiotensin II (AngII) infusion. Further studies using mice with inducible cardiomyocyte-specific STRN deletion confirmed that cardiomyocyte striatin plays a key role in this early remodelling phase.

## Methods

### Ethics statement

#### Human heart samples

Human heart samples were from the University of Pittsburgh, U.S.A. Failing human heart samples were from patients who consented to a protocol reviewed and approved by the University of Pittsburgh Institutional Review Board. Non-failing heart samples were collected under University of Pittsburgh CORID #451 (Committee for Oversight of Research and Clinical Training Involving Decedents) and with consent being obtained by the local Organ Procurement Organization (OPO), CORE (Center for Organ Recovery and Education).

#### Mouse studies

Mice were housed at the BioResource Unit at University of Reading (colonies for STRN and STRN3 global knockout) or St. George’s University of London (colonies for cardiomyocyte-specific deletion of STRN), both UK registered with a Home Office certificate of designation. Procedures were performed in accordance with UK regulations and the European Parliament Directive 2010/63/EU for animal experiments. All work was undertaken in accordance with local institutional animal care committee procedures at the University of Reading and the U.K. Animals (Scientific Procedures) Act 1986. Studies were conducted under Project Licences 70/8248, 70/8249 and P8BAB0744.

### Studies of striatin isoforms in human hearts

mRNA expression of *STRN* (ENSG00000115808), *STRN3* (ENSG00000196792) and *STRN4* (ENSG00000090372) was determined using a previously published RNASeq dataset derived from left ventricular samples of patients with end-stage dilated cardiomyopathy (*n*=97) taken at the time of transplantation or left ventricular assist device implantation, compared with non-diseased controls (*n*=108) [31]. Differential expression analysis was performed with DESeq2 (V*1.18.1*, Wald test) [32].

Human heart samples used in this study were previously used to study RAF kinases [33]. Transmural tissue at the level of the anterior papillary muscle was collected at the time of cardiac transplantation from the left ventricle of end-stage heart failure patients. Samples were collected in the operating room and transported in ice-cold St. Thomas’ cardioplegia solution, flash frozen within 20 min of excision, and stored at −80°C prior to utilisation. Control left ventricular tissues were collected from hearts that were rejected for transplant for varying reasons. Tissues were collected and stored in a similar manner as the failing hearts, with between 20 and 45 min of time elapsing between cross-clamp and freezing of the tissue. Hearts were ground to powder under liquid N_2_, and samples taken for RNA and protein preparation as described below.

### Animal husbandry and randomisation

Housing conditions were as described in [33,34]. Animals were checked daily and breeding was conducted with mice between 6 weeks and 8 months with a maximum of 6 litters per female. Mice undergoing procedures were monitored using a score sheet and routinely culled if they reached a predefined endpoint agreed with the Named Veterinary Surgeon. Weights were taken before, during and at the end of the procedures. Mouse weights from the start and end of procedures are provided in Supplementary Table S1. These studies used only male mice because of the intention to convert the line for conditional gene deletion using tamoxifen (see below), an approach which has not been fully characterised for female mice. Furthermore, our recent studies of inducible deletion of BRAF indicated that males and females responded very differently to this regime [35]. Mice were allocated to specific groups on a random basis with randomisation performed independently of the individual leading the experiment. Four mice receiving AngII died and all data for these were excluded from analysis (one wild-type from the STRN colony that died on day 2, two STRN^fl/fl^/Cre^MCM/−^ mice treated with corn-oil that died on day 7 and one STRN^fl/fl^/Cre^MCM/−^ mouse treated with tamoxifen that died on day 7). Post-mortem analysis showed rupture of a major blood vessel in all cases. Otherwise, no mice were excluded after randomisation. Individuals conducting the studies were not blinded to experimental conditions for welfare monitoring purposes. Data and sample analysis (e.g., echocardiography and histology) were performed by individuals who were blinded to intervention.

### Mouse lines and gene deletion strategy

Mice for STRN or STRN3 knockout were from the Knockout Mouse Project (KOMP). Both lines (“Knockout first” STRN^tm1a(KOMP)WTsi^ and STRN3^tm1a(KOMP)WTsi^) were on a C57Bl/6N background and, following resuscitation, were backcrossed with C57Bl/6J mice (Charles River Laboratories) for at least eight generations prior to experimentation and sperm preservation (at the Mary Lyon Centre, MRC Harwell, UK). Colonies were maintained as heterozygotes with ongoing breeding with C57Bl/6J mice to generate heterozygote and wild-type (WT) littermates for experiments (N.B. Global homozygous knockout of any striatin isoform is embryonic lethal).

*Myh6*-MERCreMER mice expressing tamoxifen-inducible Cre recombinase under control of a mouse *Myh6* promoter [Tg(Myh6-cre)1Jmk/J, strain no. 009074] [36] were from Jackson Laboratories, imported into the UK and transported to St. George’s University of London for breeding in-house. These mice are on a C57Bl/6J background. Mice for cardiomyocyte-specific knockout of STRN were derived from the sperm banked at the Mary Lyon Centre from the STRN mice. The line was resuscitated and allele conversion using FLP recombinase to generate the conditional ready floxed line was performed by the Mary Lyon Centre (MRC Harwell). Heterozygous floxed STRN (STRN^WT/fl^) mice were transported to St. George's University of London and backcrossed onto a C57Bl/6J background for four generations maintaining the line as heterozygotes before generating the homozygote line (STRN^fl/fl^). They were then crossed with homozygous Cre (Cre^+/+^) mice to generate mice that were heterozygous STRN and hemizygous for Cre (STRN^WT/fl^/Cre^+/−^); these were used to generate double homozygotes (STRN^fl/fl^/Cre^+/+^). STRN^fl/fl^ mice were bred with STRN^fl/fl^/Cre^+/+^ mice to generate mice hemizyogous for Cre and homozygous for floxed STRN (STRN^fl/fl^/Cre^+/^).

Tamoxifen was dissolved in 0.25 ml ethanol which was then mixed with 4.75 ml corn oil. Male mice (8–9 weeks) were treated with a single dose of tamoxifen (40 mg/kg i.p.; Sigma-Aldrich) to induce recombination or corn-oil vehicle as a control at 4 days relative to mini-pump implantation (see below). Our previous studies of mice hemizygous for Cre^MCM/−^ treated in this way demonstrated that this is sufficient to induce recombination but has no overt effect on cardiac function or dimensions at baseline or on AngII-induced cardiac hypertrophy [33,35].

### Genotyping and confirmation of recombination

Ear notches were taken for identification using a 0.5 mm ear punch and used for genotyping. For confirmation of recombination in the heart, hearts and kidneys were collected from mice treated with tamoxifen or corn-oil vehicle, the tissues were ground to powder under liquid N_2_ and samples were taken. DNA was extracted using Purelink genomic DNA (gDNA) mini-kits (Invitrogen) according to the manufacturer’s instructions. gDNA was purified through Purelink spin columns and eluted in 30 µl of elution buffer. PCR amplification used GoTaq Hot Start Polymerase (Promega). PCR conditions were 95°C for 3 min, followed by up to 35 cycles of 95°C denaturations for 30 s, 30 s annealing, elongation at 72°C for 30 s, followed by a 7-minute 72°C final extension. Details of primers and conditions are in Supplementary Table S2. PCR products were separated using gel electrophoresis (25 min, 80 V) on 2% (w/v) agarose gels and visualised under UV light.

### AngII-induced cardiac hypertrophy

Alzet osmotic minipumps (supplied by Charles River Laboratories) were used for continuous delivery of 0.8 mg/kg/d AngII or vehicle for 7 d. Mice were anaesthetised in an induction chamber using vaporised 5% isoflurane in a constant oxygen supply of 2 l/min. Anaesthesia was maintained at 2.5% isoflurane using a nose cone. Mice were positioned on a heated mat in the prone position. Buprenorphine (Vetergesic, Ceva Animal Health Ltd.) (0.05 mg/kg, diluted in sterile PBS) was administered subcutaneously for analgesia. The fur covering the mid-scapular region was removed using an electric razor and the area was sterilised with HIBISCRUB® (VioVet). Under aseptic conditions, a 2 cm incision was made at the mid-scapular region and blunt dissection generated a pocket towards the lower-left flank of the mouse for the minipump to be inserted. The wound was closed with two simple interrupted sutures using polypropylene 4-0 thread (Prolene, Ethicon) and then sterilised with HIBISCRUB®. Mice were recovered singly and returned to a clean cage once fully recovered.

### Mouse echocardiography

Echocardiography was performed using a high-frequency ultrasound system (Vevo 2100™, Visualsonics) equipped with a 38 MHz MS400 transducer. Baseline echocardiograms were collected at 8 weeks (prior to tamoxifen treatment and/or minipump implantation) with additional scans taken at the end of the study. Mice were anaesthetised in an induction chamber using vaporised 5% isoflurane in a constant oxygen supply of 1 l/min. Anaesthesia was maintained with 1.5% isoflurane using a nose cone. Mice were positioned on a heating physiological monitoring stage in a supine position. Heart rate, respiration rate and body temperature were monitored. Chest fur was removed with an electric razor and hair removal cream. Pre-warmed ultrasound gel was applied to the chest as a coupling medium for the transducer. The transducer was orientated and lowered into the ultrasound gel until a clear image was centralised on the monitor. Imaging was completed within a maximum time of 30 min, and usually within 15 min. Mice were recovered singly and transferred to the home cage once fully recovered. Cardiac function and left ventricular wall dimensions were measured from M-mode short axis images using VevoLab software with assistance from the autoLV tool. Cardiac function and global longitudinal strain were measured from B-mode long axis images using VevoStrain software for speckle tracking. B-mode images of the ascending aorta were also captured and the diameter of the aorta measured using VevoLab software, before the beginning of the arch and perpendicular to the walls. Measurements were collected at the end of ventricular contraction when the diameter was at its largest and following contraction of the aorta when at its narrowest. At the end of the experiment, whilst still under anaesthesia, mice were culled by cervical dislocation with severance of the femoral artery to ensure cessation of life. Hearts were excised quickly, washed in PBS, dried and snap-frozen in liquid N_2_ or fixed for histology.

### Histology and analysis

Histological sections for the global STRN and STRN3 knockout mouse studies were prepared and stained by HistologiX Limited. Sections for the cardiomyocyte-specific STRN knockout study were prepared and stained at St. George’s University of London (as described in [37]). Haemotoxylin and eosin staining was used for analysis of myocyte cross‐sectional area. Cells around the periphery of the left ventricle (excluding epicardial layer) were chosen at random (ensuring that the cells were in cross-section and with a clear, rounded nucleus) and outline traced using NDP.view2 software (Hamamatsu). This approach was taken to ensure cells were captured in the same region of the myocardium, to avoid issues relating to different levels of stress and orientation of cells across the myocardial wall, along with interdigitation of individual cardiomyocytes. Because of the rigorous approach, limited numbers of cells were available for selection and all, or up to 30 cells were measured per section by a single independent assessor and the mean value taken for each mouse. To assess interstitial fibrosis, sections were stained with Masson’s trichrome or picrosirius red and analysis used Image-J as in [37]. The collagen fraction was calculated as the ratio between the sum of the total area of fibrosis (blue colour for Masson’s trichrome, red colour for picrosirius red) to the sum of the total tissue area (including the myocyte area) for the entire image and expressed as a percentage. For perivascular fibrosis (because there was not a constant number of vessels apparent in each section), picrosirius red staining was used and the whole section was scored for perivascular fibrosis around arterioles (identified by a clear elastic layer). Values were 1 (negligible increase in fibrosis around any vessel), 2 (mild-to-moderate fibrosis around 1 or more vessels), 3 (significant fibrosis permeating tissue around 1 or more vessels) and 4 (extensive fibrosis around multiple vessels, penetrating into the myocardium).

### RNA preparation and qPCR

Heart powders (10–15 mg) were weighed into safelock Eppendorf tubes and kept on dry ice. RNA Bee (AMS Biotechnology Ltd.) was added (1 ml) and the samples homogenised on ice using a pestle. RNA was prepared according to the manufacturer’s instructions and dissolved in nuclease-free water. The purity was assessed from the *A*_260_/*A*_280_ measured using an Implen NanoPhotometer (values were 1.8–2.0) and concentrations determined from the *A*_260_. Quantitative PCR (qPCR) analysis was performed as described in [38]. Total RNA was reverse transcribed to cDNA using High Capacity cDNA Reverse Transcription Kits with random primers (Applied Biosystems). qPCR was performed using a StepOnePlus Real-Time PCR system (ThermoFisher Scientific) using 1/40 of the cDNA produced. Optical 96-well reaction plates were used with iTaq Universal SYBR Green Supermix (Bio-Rad Laboratories Inc.) according to the manufacturer's instructions. See Supplementary Table S3 for primer sequences. Results were normalised to *GAPDH*, and relative quantification was obtained using the ΔCt (threshold cycle) method; relative expression was calculated as 2^−ΔΔCt^, and normalised as indicated in the Figure Legends.

### Immunoblotting

Heart powders (15–20 mg) were homogenised in 6 vol extraction buffer [20 mM Tris pH 7.5, 1 mM EDTA, 10% (v/v) glycerol, 1% (v/v) Triton X-100, 100 mM KCl, 5 mM NaF, 0.2 mM Na_3_VO_4_, 5 mM MgCl_2_, 0.05% (v/v) 2-mercaptoethanol, 10 mM benzamidine, 0.2 mM leupeptin, 0.01 mM trans-epoxy succinyl-L-leucylamido-(4-guanidino)butane, 0.3 mM phenylmethylsulphonyl fluoride, 4 µM microcystin]. Samples were extracted on ice with intermittent vortex mixing (10 min), then centrifuged (10,000 × ***g***, 10 min, 4°C) to pellet insoluble material. The supernatants were removed, a sample was taken for protein assay and the rest boiled with 0.33 vol sample buffer (300 mM Tris-HCl pH 6.8, 10% (w/v) SDS, 13% (v/v) glycerol, 130 mM dithiothreitol, 0.2% (w/v) bromophenol blue). Protein concentrations were determined by BioRad Bradford assay using a 1/5 dilution (v/v) in H_2_O of the dye reagent concentrate and BSA standards.

Proteins (100 µg for human heart samples, 40 µg for rat and mouse heart samples) were separated by SDS-PAGE (200 V) using 8% (for striatin isoforms), or 12% (GAPDH) polyacrylamide resolving gels with 6% stacking gels until the dye front reached the bottom of the gel (∼50 min). Proteins were transferred electrophoretically to nitrocellulose using a BioRad semi-dry transfer cell (10 V, 60 min). Non-specific binding sites were blocked (15 min) with 5% (w/v) non-fat milk powder in Tris-buffered saline (20 mM Tris-HCl pH 7.5, 137 mM NaCl) containing 0.1% (v/v) Tween 20 (TBST). Blots were incubated with primary antibodies in TBST containing 5% (w/v) BSA (overnight, 4°C), then washed with TBST (3 × 5 min, 21°C), incubated with horseradish peroxidase-conjugated secondary antibodies in TBST containing 1% (w/v) non-fat milk powder (60 min, 21°C) and then washed again in TBST (3 × 5 min, 21°C). Rabbit polyclonal antibodies to STRN and STRN4 were from Novus Biologicals Ltd (STRN: catalogue number NB110-74571; STRN4: catalogue no. NBP2-36537) and were used at 1/1000 dilution. Goat polyclonal antibodies to STRN3 (SG2NA) were from Santa Cruz Biotechnology Inc (catalogue no. E1704). Rabbit polyclonal antibodies to GAPDH were from Cell Signaling Technologies (catalogue no. 14C10). All primary antibodies were used at 1/1000 dilutions. Horseradish-peroxidase-conjugated goat anti-rabbit immunoglobulins (catalogue no. P0448) and rabbit anti-goat immunoglobulins (catalogue no. P0449) were from Dako (supplied by Agilent) and were used at 1/5000 dilution. Bands were detected by enhanced chemiluminescence using ECL Prime with visualisation using an ImageQuant LAS4000 system (Cytiva). ImageQuant TL 8.1 software (GE Healthcare) was used for densitometric analysis. Raw values for phosphorylated kinases were normalised to the total kinase. Values for all samples were normalised to the mean of the controls.

### Image processing and statistical analysis

Images were exported from the original software as .tif or .jpg files and cropped for presentation with Adobe Photoshop CC maintaining the original relative proportions. Data analysis used Microsoft Excel and GraphPad Prism 9. Statistical analysis was performed using GraphPad Prism 9. A Grubb’s outlier test was applied to the data, and outliers excluded from the analysis. Statistical significance was determined using two-tailed unpaired Mann–Whitney tests, or two-tailed one-way or two-way ANOVA as indicated in the Figure Legends. A Holm-Sidak’s multiple comparison test was used in combination with ANOVA. Graphs were plotted with GraphPad Prism 9 or 10. Specific *P* values are provided with significance levels of *P*<0.05 in bold type.

## Results

### Striatin and striatin 3 are up-regulated in human failing hearts

**Figure 1:**
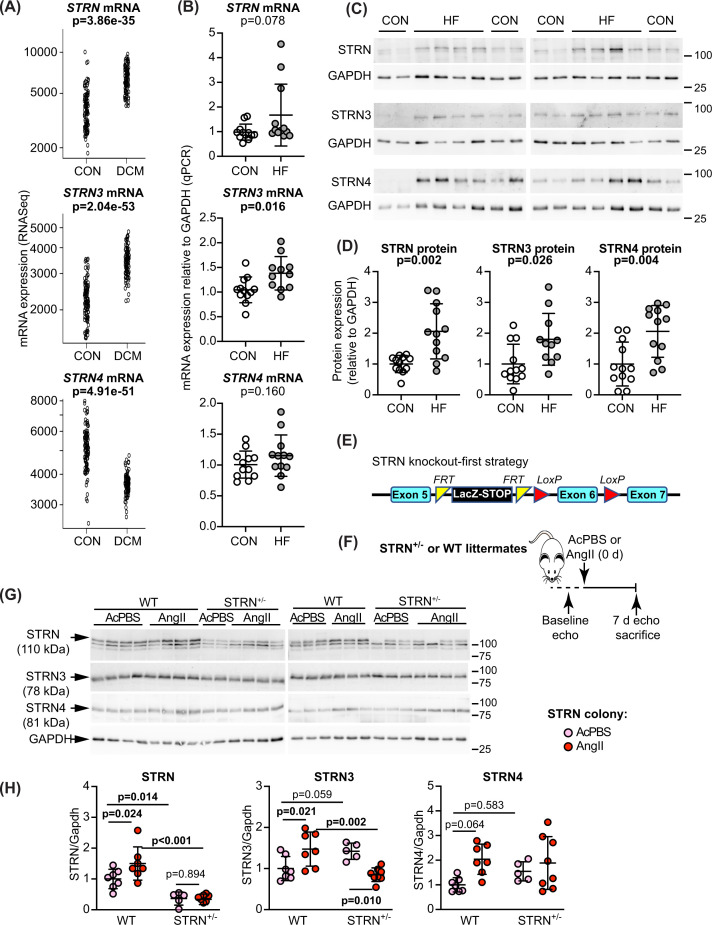
Expression of striatin isoforms in human failing hearts and global heterozygous STRN knockout in mice (**A**) Data for mRNA expression of striatin isoforms in human hearts were from an RNASeq database of patients with dilated cardiomyopathy (DCM, *n*=97) and normal controls (CON, *n*=108). Data for individual samples are shown with adjusted *P* values. (**B–D**) Samples from control hearts (CON) or explanted hearts from patients with heart failure (HF) were used to prepare RNA for qPCR analysis (B) or protein for immunoblots (C,D). Representative immunoblots (100 µg protein per lane) are in (C) with densitometric analysis in (D). Individual values are shown with means ± SD. Results are relative to GAPDH and normalised to the mean of the CON hearts. Mann–Whitney tests were used for statistical analysis. (**E**) ‘Knockout-first’ strategy for global deletion of STRN in mice involved positioning of a STOP cassette flanked by FRT sites upstream of a critical exon that was also flanked with LoxP sites. (**F**) Experimental approach for assessment of effects of STRN deletion on cardiac function. Homozygous global knockout of STRN is embryonic lethal, so heterozygote STRN^+/−^ male mice (8 weeks) were used in comparison with wild-type (WT) littermates from each colony. Following baseline echocardiography (echo), minipumps were implanted for delivery of acidified PBS vehicle (AcPBS) or 0.8 mg/kg/d angiotensin II (AngII). Following echocardiography at 7 d, mice were killed. (**G,H**) Heart powders were used for immunoblotting (40 µg protein per lane). Representative immunoblots of the striatin isoforms and GAPDH (G) are shown with densitometric analysis (H). Results are relative to GAPDH and normalised to the means for WT mice treated with AcPBS. N.B.: The upper band of the STRN blot used for densitometry correlates with the predicted molecular weight of STRN protein (110 kDa).

To assess which of the striatin isoforms is most likely to promote human heart failure, we mined an RNASeq database of heart samples from patients with dilated cardiomyopathy (DCM; *n* =97) compared with normal controls (*n*=108) [31]. *STRN*, *STRN3* and *STRN4* transcripts were readily detected, with a rank order of expression of *STRN4*>*STRN*>*STRN3* in control samples (Figure 1A). Expression of STRN4 declined in DCM hearts whilst expression of *STRN* and *STRN3* increased. To establish if this reflects a broader spectrum of heart failure and determine how mRNA expression correlates with protein expression, we also assessed expression of striatins in samples from 12 patients with heart failure of mixed non-ischaemic aetiology compared with normal controls (previously reported in [33]). These showed a significant increase in only *STRN3* mRNA expression in heart failure samples (Figure 1B), although protein expression of all striatins was significantly increased in failing hearts (Figure 1C,D). Thus, although STRN and STRN3 have a similar profile overall with up-regulation in disease, STRN4 may have more specific and selective roles in different forms of heart failure.

### Global heterozygous knockout of STRN in mice, but not STRN3, reduces cardiac hypertrophy induced by AngII

Since STRN and STRN3 were up-regulated in DCM and human failing hearts of other aetiology, we focused on these isoforms for further investigation. We used commercially-available knockout-first mice, engineered with a removable STOP cassette to permit conversion for conditional gene deletion (Figure 1E; Supplementary Figure S1A). Homozygous global deletion of any striatin isoform is embryonic lethal, so we assessed the effects of heterozygous gene deletion (STRN^+/−^ and STRN3^+/−^), comparing responses with wild-type littermates. We studied male mice taking baseline echocardiograms at 8 weeks and, using M-mode assessment of short axis images, or B-mode images with speckle-tracking/strain analysis, we detected no differences in cardiac function or dimensions in STRN^+/−^ or STRN3^+/−^ mice compared with the wild-types (Supplementary Table S4). Mice were then treated for 7 days with acidified PBS (AcPBS) vehicle or with 0.8 mg/kg/d AngII using osmotic minipumps (Figure 1F). This ‘slow-pressor’ dose gradually induces hypertension over 7–14 days [39–41]. We did not assess the effects in female mice because premenopausal mice are resistant to AngII-induced hypertension [42,43] (see Discussion).

We assessed protein expression of the three striatins in mouse hearts by immunoblotting. The only antibody for STRN that we could identify detected STRN protein in mouse hearts as a band of ∼110 kDa (the upper band on the immunoblots), and this was reduced in hearts from STRN^+/−^ mice (Figure 1G,H). Other bands were detected below this but, since there is no evidence for alternatively spliced isoforms of STRN and we detected no difference in expression of these bands in the STRN^+/−^ mice, we assume these are non-specific. STRN knockdown did not significantly affect expression of STRN3 or STRN4. AngII increased expression of STRN in wild-type littermates from the STRN colony, and this was accompanied by a significant increase in STRN3. STRN3 was reduced in hearts from STRN3^+/−^ mice with no significant change in expression of STRN or STRN4 (Supplementary Figure 1C,D). In contrast to the STRN colony, we did not detect an increase in any striatin isoform with AngII in hearts from wild-type mice from the STRN3 colony, presumably reflecting differences in the background strain despite extensive backcrossing onto the same C57Bl/6J background.

**Figure 2:**
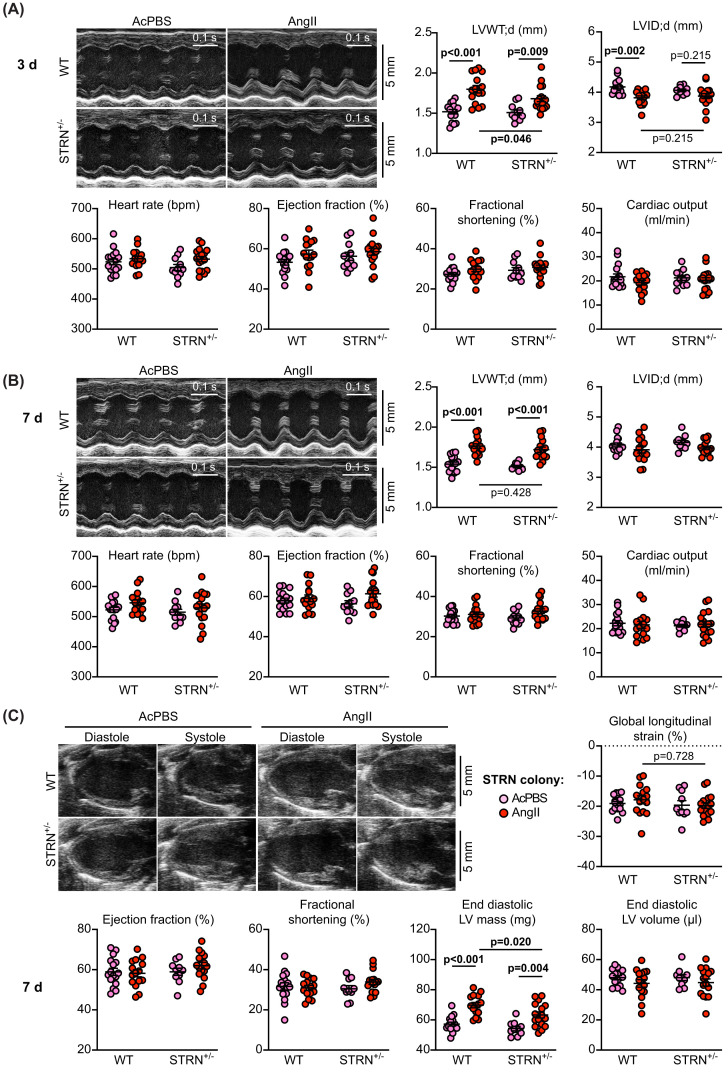
Heterozygous global deletion of STRN compromises the hypertrophic response to AngII Male mice (8 wks) heterozygote for STRN knockout (STRN^+/−^) and wild-type (WT) littermates from the same colony were treated with acidified PBS (AcPBS) vehicle or AngII (0.8 mg/kg/d). Cardiac function and dimensions were assessed by echocardiography using M-mode imaging of the short axis at 3 d (**A**) or 7 d (**B**), or B-mode imaging of the long axis at 7 d with speckle-tracking and strain analysis (**C**). For M-mode imaging, diastolic values for left ventricle (LV) wall thickness (WT) or internal diameter (ID) are shown and end diastolic LV mass and volume are provided for B-mode imaging. Cardiac function measurements are shown for M-mode and B-mode analysis for comparison. Representative images are in the upper left of each panel. Individual datapoints are plotted with means ± SEM. Statistical analysis used two-way ANOVA with Holm-Sidak’s post-test. N.B.: All echocardiography data are provided in Supplementary Tables S4, S5 and S7.

We determined the effects of AngII on STRN^+/−^ and STRN3^+/−^ mouse hearts using echocardiography, assessing the changes induced at 3 and 7 d after minipump implantation. We analysed short axis M-mode images to obtain information on cardiac function and left ventricle dimensions (wall thickness and internal diameter) as is usual, but also employed the newer modality of speckle-tracking/strain analysis of long axis B-mode images. The latter offers a significant advantage in monitoring changes around the entire ventricle wall rather than across a single point in the myocardium. For cardiac function (heart rate, ejection fraction, fractional shortening and cardiac output), we obtained similar measurements with either M-mode analysis or B-mode speckle-tracking software (Figure 2; Supplementary Figure S2 and Supplementary Tables S5–7). We detected no significant changes in cardiac function with AngII treatment in STRN^+/−^ and STRN3^+/−^ mouse hearts or their wild-type littermates.

M-mode analysis was used to assess wall thickness and internal diameter at the level of the papillary muscle across the left ventricle. AngII induced a significant increase in left ventricle wall thickness in wild-type mice from both STRN^+/−^ and STRN3^+/−^ colonies at 3 d, together with a decrease in internal diameter, consistent with concentric hypertrophy (Figure 2; Supplementary Figure S2 and Supplementary Tables S5–7). Wall thickness was also increased in STRN^+/−^ mice treated with AngII, but this was significantly less than in the wild-type littermates (Figure 2A). By 7 d, the increase in wall thickness induced by AngII in STRN^+/−^ mice was no longer significantly different from the wild-type littermates (Figure 2B), presumably a consequence of further remodelling of the heart. B-mode imaging confirmed this was likely to be the case since AngII induced a significant increase in estimated left ventricle mass in wild-type mice with a significantly reduced overall response in STRN^+/−^ mice (Figure 2C). This was not associated with any differences in global longitudinal strain between wild-type and STRN^+/−^ mice which, along with the absence of change in cardiac functional measurements, indicated that the hypertrophic response was still in a compensatory phase. In contrast to STRN^+/−^ mice, the responses to AngII of STRN3^+/−^ mouse hearts were similar to those of wild-type littermates with increases in left ventricle wall thickness (using M-mode analysis) and estimated left ventricle mass (with B-mode speckle-tracking) (Supplementary Figure S2). This was accompanied by a small increase in global longitudinal strain suggesting there could be some gain of contractile function but, together with the standard measures of cardiac function, the data indicate that these hearts were also in a compensatory phase.

**Figure 3:**
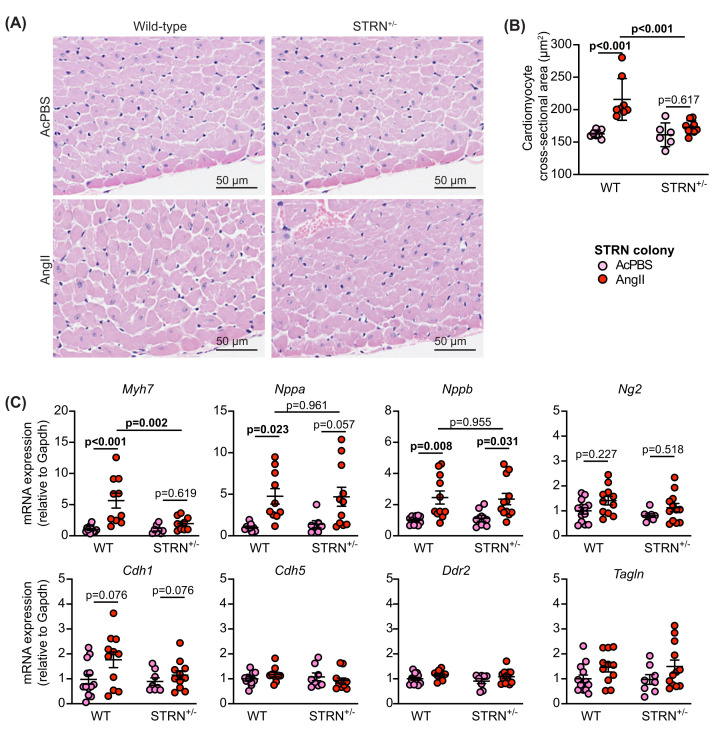
Heterozygous global deletion of STRN, reduces cardiomyocyte hypertrophy and the increase in expression of *Myh7* induced by AngII 8 wk male STRN^+/−^ mice, plus their wild-type (WT) littermates were treated with acidified PBS (AcPBS) vehicle or 0.8 mg/kg/d AngII (7 d). Hearts were fixed and sections stained with haemotoxylin and eosin. Representative images (**A**) show areas from the outer perimeter of the left ventricular wall opposite the interventricular septum. (**B)** Cardiomyocyte cross-sectional areas are shown. (**C**) RNA was extracted from mouse heart powders and analysed by qPCR for the mRNAs shown. Individual datapoints are plotted with means ± SEM. Results are relative to GAPDH and normalised to the means for WT mice treated with AcPBS. Statistical analysis used two-way ANOVA with Holm-Sidak’s post-test.

Heart sections were stained with haemotoxylin and eosin and the cross-sectional area of myocytes at the periphery of the left ventricle measured. AngII increased cardiomyocyte cross-sectional area (indicative of cardiomyocyte hypertrophy) in wild-type mice from both STRN and STRN3 colonies (Figure 3A,B and Supplementary Figure S3A,B), and increased expression of hypertrophic gene markers (Figure 3C and Supplementary Figure S3C). The AngII-induced increase in cross-sectional area and expression of *Myh7* mRNA (though not *Nppa* or *Nppb*) was reduced in hearts from STRN^+/−^ mice (but not STRN3^+/−^ mice) compared with their wild-type littermates. Other genes associated with cardiac non-myocytes (*Ng2* for pericytes, *Tagln* for smooth muscle cells, *Ddr2* for fibroblasts, *Cdh1* and *Cdh5* for endothelial cells) were not significantly changed with AngII treatment or between wild-type and STRN^+/−^ mice, suggesting the responses were not associated with significant changes in the proportions of cardiac cell types at this stage (Figure 3C).

**Figure 4:**
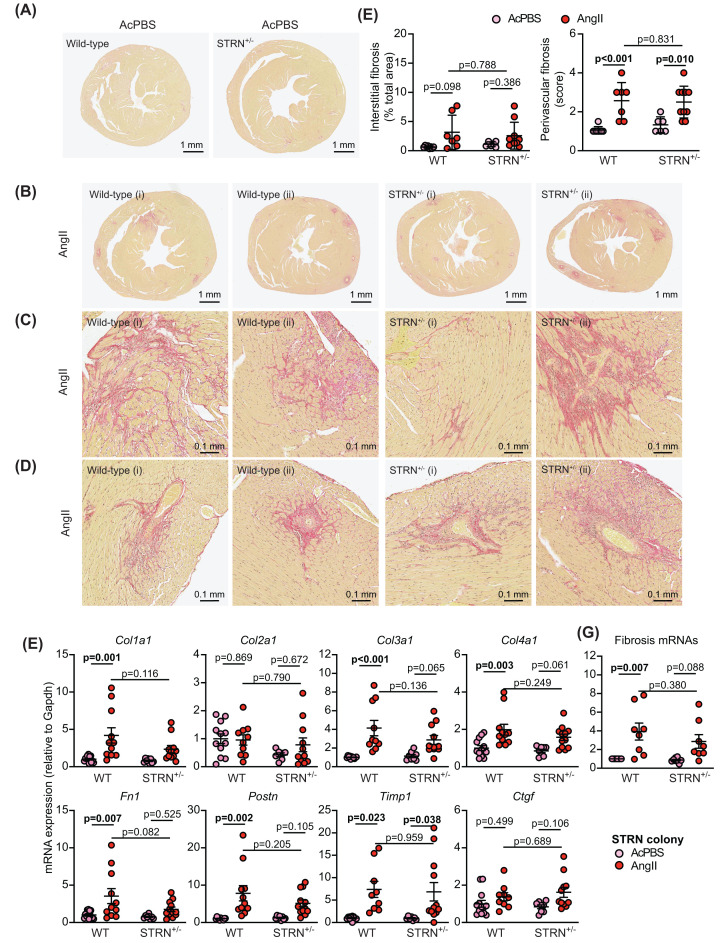
Heterozygous knockout of STRN does not reduce cardiac fibrosis induced by AngII Eight-week male STRN^+/−^ mice, plus their wild-type (WT) littermates were treated with acidified PBS (AcPBS) vehicle or 0.8 mg/kg/d AngII (7 d). Hearts were fixed and sections stained with picrosirius red. Representative short axis views of the whole heart are shown for wild-type and STRN^+/−^ mice treated with AcPBS (**A**) or AngII (**B**). For AngII-treated hearts, an average (i) or maximum (ii) response is shown. Enlarged regions of the AngII-treated hearts in (B) are shown below for interstitial fibrosis (**C**) or perivascular fibrosis (**D**). (**E**) Interstitial fibrosis was measured using ImageJ and is presented as the % of the total area (excluding regions around the blood vessels). Perivascular fibrosis was scored (1: negligible increase in fibrosis around any vessel; 2: mild-to-moderate fibrosis around 1 or more vessels; 3: Significant fibrosis permeating tissue around 1 or more vessels; 4: extensive fibrosis around multiple vessels, penetrating into the myocardium). A scoring system was used for the latter because of the variation in numbers of vessels seen in different heart sections. (**F**) RNA was extracted from mouse heart powders and analysed by qPCR for fibrosis mRNAs as indicated. (**G**) The average value for each condition for each of the genes shown in (**F**) was taken. qPCR results are relative to GAPDH and normalised to the means for WT mice treated with AcPBS. Individual datapoints are plotted with means ± SEM. Statistical analysis used two-way ANOVA with Holm-Sidak's post-test.

Heart sections were stained with picrosirius red to assess cardiac fibrosis (Figure 4A–E and Supplementary Figure S3D–E). At this 7-day time point, AngII induced only a small, non-significant increase in interstitial fibrosis and this was most commonly detected in the area at the junction between the interventricular septum and the ventricular wall. However, there was a greater, significant increase in perivascular fibrosis. The degree of fibrosis induced by AngII was similar in STRN^+/−^, STRN3^+/−^ and their wild-type littermates. AngII also upregulated mRNAs encoding fibrotic genes (Figure 4F,G). This was not significantly different in STRN^+/−^ or STRN3^+/−^ mouse hearts compared with their wild-type littermates, but there was an indication of reduced expression of some genes in the STRN^+/−^ mouse hearts.

Overall, the data indicate that reduction of STRN3 does not substantially affect cardiac hypertrophy induced by AngII, at least over the short term. In contrast, reduction of STRN compromises the cardiac response to AngII, having a clear effect on cardiomyocyte hypertrophy, though not cardiac fibrosis.

### Cardiomyocyte-specific deletion of STRN inhibits AngII-induced cardiac hypertrophy

**Figure 5:**
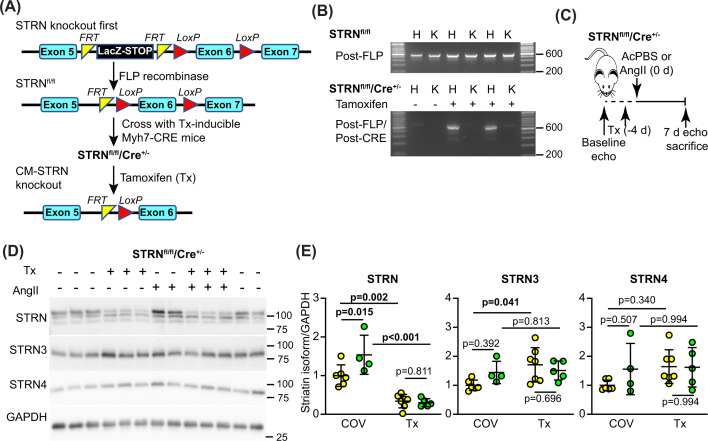
Cardiomyocyte-specific knockout of STRN inhibits cardiac hypertrophy induced by AngII (**A**) Strategy for cardiomyocyte (CM) specific knockout of STRN in mice. STRN knockout first mice were converted to ‘conditional-ready’ using FLP recombinase, removing the STOP cassette between exons 5 and 6, whilst leaving the LoxP sites surrounding exon 6 in place (STRN^fl/fl^ mice). These were bred with mice expressing tamoxifen- (Tx-) activated Cre to generate mice homozygous for floxed striatin and hemizygous for Cre (STRN^fl/fl^/Cre^+/−^) for experiments. Treatment with tamoxifen induced recombination and deletion of exon 6. (**B**) Hearts (H) and kidneys (K) from male mice were genotyped to confirm that the mice were conditional-ready (upper panel) and that tamoxifen treatment (40 mg/kg) induced recombination in the heart but not kidney (lower panel). (**C**) Strategy for experiments. Male STRN^fl/fl^/Cre^+/−^ mice (8 weeks) were used. Following baseline echocardiography (echo), mice were treated with corn-oil vehicle (COV) or Tx in COV (40 mg/kg; day -4) and minipumps were implanted (day 0) to deliver acidified PBS (AcPBS) vehicle or 0.8 mg/kg/d AngII for 7 days, after which a final echocardiogram was taken before the mice were sacrificed. (**D,E**) Heart powders were used for immunoblotting (40 µg protein per lane). Representative immunoblots of the striatin isoforms and GAPDH are in (D) with densitometric analysis in (E). Results are relative to GAPDH and normalised to the means for mice treated with vehicle only. The upper band of the STRN blot used for densitometry correlates with the predicted molecular weight of STRN protein.

To determine if the reduced hypertrophic response to AngII in STRN^+/−^ mice was due to reduced expression of STRN in cardiomyocytes rather than other cardiac cells, we converted the STRN knockout-first line for conditional gene deletion using FLP recombinase (Figure 5A). These mice were used to generate homozygous floxed STRN mice with a single allele for tamoxifen-inducible Cre under the control of a Myh6 promoter [36]. Male STRN^fl/fl^/Cre^+/−^ mice (8 weeks) were treated with a single dose of tamoxifen (40 mg/kg) to induce recombination, an approach which is not associated with significant cardiotoxicity from the Cre enzyme [33,35]. Recombination was detected in hearts but not kidneys from STRN^fl/fl^/Cre^+/−^ mice treated with tamoxifen (Figure 5B), confirming cardiac-specific gene deletion. Osmotic minipumps were implanted 4 days after tamoxifen treatment (by which time the tamoxifen has been cleared from the body [44]) to deliver acidified PBS vehicle or 0.8 mg/kg/d AngII for 7 days. Immunoblotting confirmed that tamoxifen induced a significant and substantial decrease in STRN expression in the hearts of STRN^fl/fl^/Cre^+/−^ mice, and this was associated with a significant increase in expression of STRN3 (Figure 5D,E). As with global heterozygous STRN^+/−^ mice (Figure 1F,G), AngII increased expression of STRN, but there was no increase in the hearts of cardiomyocyte STRN knockout mice (Figure 5D,E).

**Figure 6:**
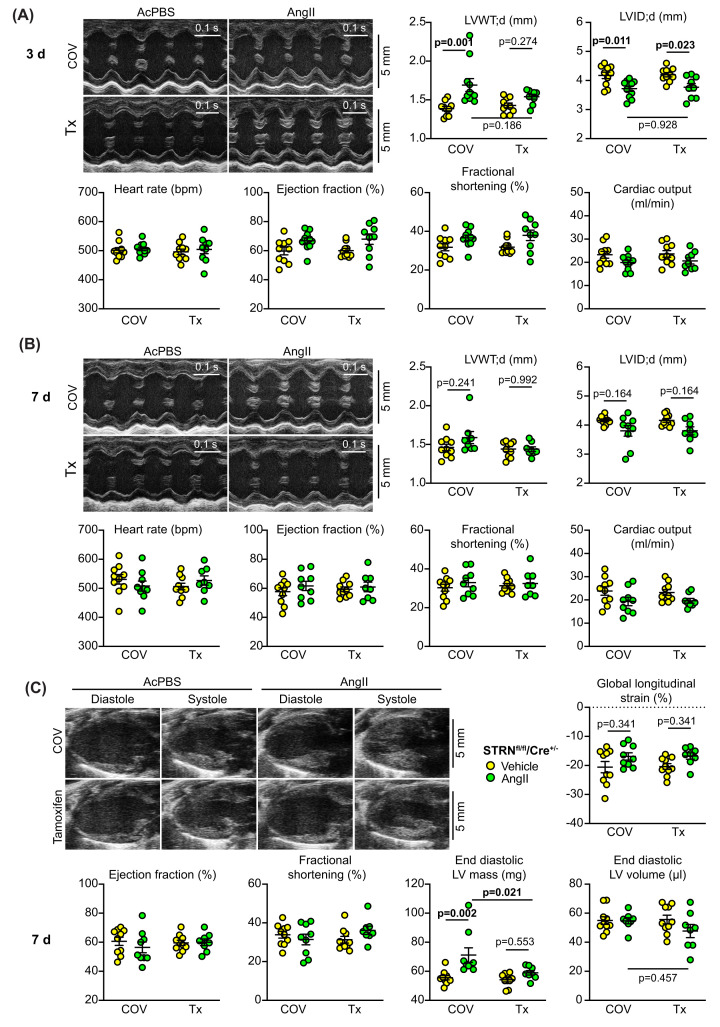
Cardiomyocyte-specific knockout of STRN compromises the hypertrophic response to AngII Male STRN^fl/fl^/Cre^+/−^ mice (8 weeks) were treated with corn-oil vehicle (COV) or tamoxifen (Tx; 40 mg/kg) and minipumps implanted to deliver acidified PBS (AcPBS) or 0.8 mg/kg/d AngII for 7 days. Cardiac function and dimensions were assessed by echocardiography using M-mode imaging of the short axis at 3 d (**A**) or 7 d (**B**), or B-mode imaging of the long axis at 7 d with speckle-tracking and strain analysis (**C**). For M-mode imaging, diastolic values for left ventricle (LV) wall thickness (WT) or internal diameter (ID) are shown and end diastolic LV mass and volume are provided for B-mode imaging. Cardiac function measurements are shown for both M-mode and B-mode for comparison. Individual datapoints are plotted with means ± SEM. Statistical analysis used two-way ANOVA with Holm-Sidak’s post-test. N.B.: All echocardiography data are provided in Supplementary Tables S9 and S10.

We used echocardiography to assess the changes in cardiac function and dimensions induced by AngII 3 and 7 d after minipump implantation in mice with and without cardiomyocyte STRN knockout (Figure 6 and Supplementary Tables S8,9). As with global heterozygous STRN gene deletion, we detected no significant differences in cardiac function between any of the conditions at either of the times studied using either M-mode or B-mode analysis. AngII induced a significant increase in left ventricle wall thickness and decrease in internal diameter after 3 d in mice without cardiomyocyte STRN knockout as assessed by M-mode imaging, but there was no increase in wall thickness in mice with cardiomyocyte STRN knockout. As with the STRN^+/−^ colony, this hypertrophy was no longer detectable at 7 d using M-mode imaging. However, using B-mode imaging and speckle-tracking for the entire wall of the left ventricle, at 7 d, AngII induced a significant overall increase in estimated left ventricle mass in mice with cardiomyocyte STRN that was lost with cardiomyocyte STRN knockout (Figure 6C).

**Figure 7:**
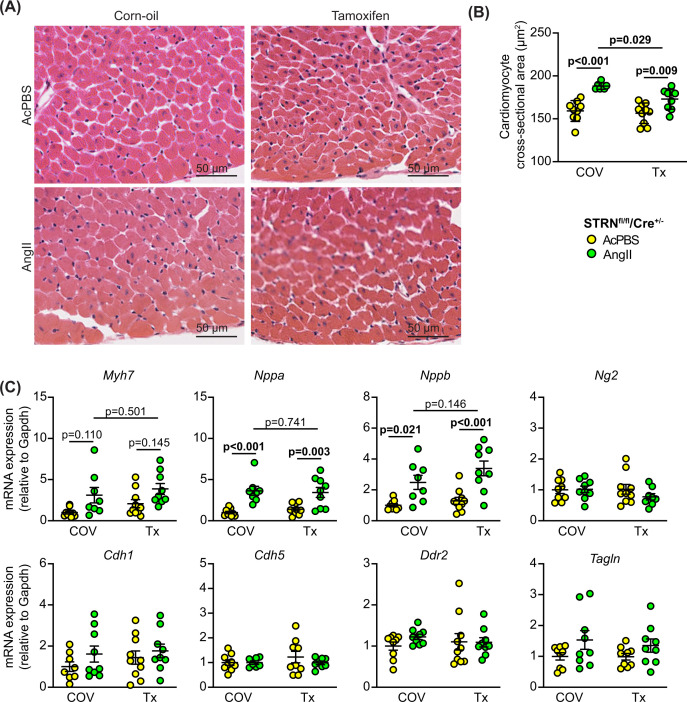
Cardiomyocyte-specific knockout of STRN inhibits the increase in cardiomyocyte cross-sectional area induced by AngII Male STRN^fl/fl^/Cre^+/−^ mice (8 wks) were treated with corn-oil vehicle (COV) or tamoxifen (Tx; 40 mg/kg) and minipumps implanted to deliver acidified PBS (AcPBS) or 0.8 mg/kg/d AngII for 7 days. Hearts were fixed or snap-frozen in liquid N_2_ before grinding to powder. Representative images of sections stained with haemotoxylin and eosin (**A**) show areas from the outer perimeter of the left ventricular wall opposite the interventricular septum. (**B**) Cardiomyocyte cross-sectional areas. (**C**) RNA extracted from mouse heart powders was analysed by qPCR for the mRNAs shown. Results are relative to GAPDH and normalised to the means for mice treated with vehicle only.

**Figure 8:**
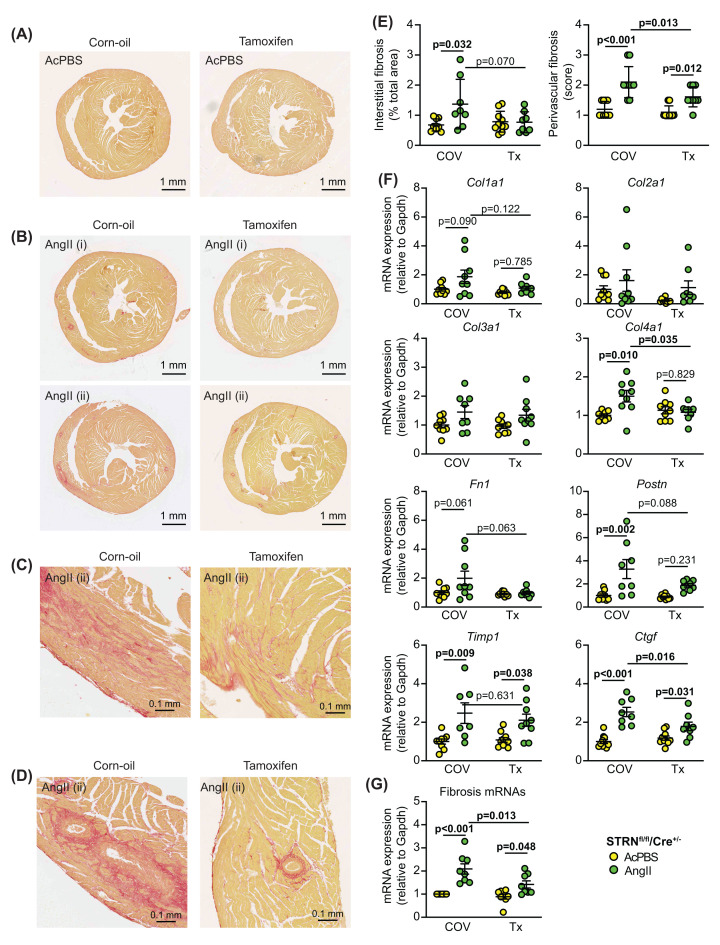
Cardiomyocyte-specific knockout of STRN inhibits the increase in cardiac fibrosis induced by AngII Male STRN^fl/fl^/Cre^+/−^ mice (8 wks) were treated with corn-oil vehicle (COV) or tamoxifen (Tx; 40 mg/kg) and minipumps implanted to deliver acidified PBS (AcPBS) or 0.8 mg/kg/d AngII for 7 days. Hearts were fixed and sections stained with picrosirius red. Representative short axis views of the whole heart are shown for mice treated with AcPBS (**A**) or AngII (**B**). For AngII-treated hearts, the average (i) or maximum (ii) response is shown. Enlarged regions of the AngII-treated hearts in (B) are shown below for interstitial fibrosis (**C**) or perivascular fibrosis (**D**). (**E**) Interstitial fibrosis was measured using ImageJ and is presented as the % of the total area (excluding regions around the blood vessels). Perivascular fibrosis was scored (1: negligible increase in fibrosis around any vessel; 2: mild to moderate fibrosis around 1 or more vessels; 3: Significant fibrosis permeating tissue around 1 or more vessels; 4: extensive fibrosis around multiple vessels, penetrating into the myocardium). A scoring system was used for the latter because of the variation in numbers of vessels seen in different heart sections. (**F**) RNA was extracted from mouse heart powders and analysed by qPCR for fibrosis mRNAs as indicated. (**G**) The average value for each condition for each of the genes shown in (**F**) was taken. qPCR results are relative to GAPDH and normalised to the means for WT mice treated with AcPBS. Individual datapoints are plotted with means ± SEM. Statistical analysis used two-way ANOVA with Holm-Sidak’s post-test.

AngII increased cardiomyocyte cross-sectional area and this was significantly reduced with cardiomyocyte STRN knockout (Figure 7A,B). However, hypertrophic gene marker expression (*Myh7*, *Nppa* and *Nppb*) was similar with or without tamoxifen treatment (Figure 6C) suggesting the cells continued to undergo pathological stress. As with the STRN*^+/-^* colony, gene markers for cardiac non-myocytes showed no significant change in expression. In contrast with the effect of global heterozygous STRN knockout (Figure 4A–C), the increase in interstitial and perivascular fibrosis induced by AngII was significantly inhibited with cardiomyocyte STRN knockout and this was associated with reduced expression of fibrotic gene markers (Figure 8). We conclude that cardiomyocyte STRN plays an important role in early adaptive remodelling of the heart induced by AngII, with effects at the level of the cardiomyocytes themselves to promote hypertrophic growth and to increase cardiac fibrosis.

**Figure 9:**
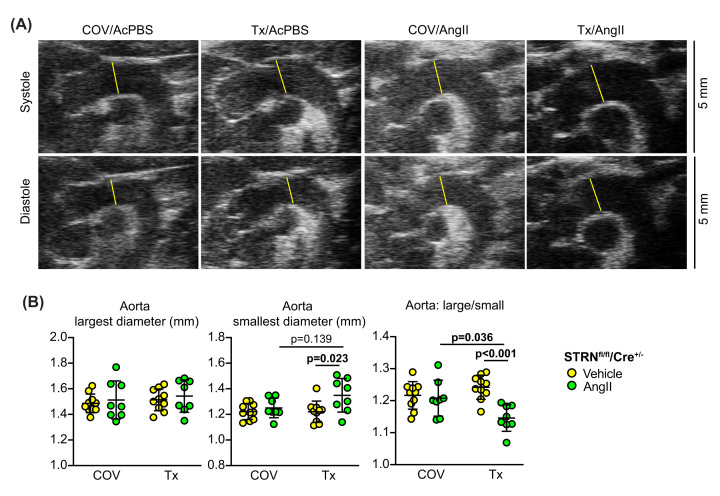
Loss of aortic elasticity in mice with cardiomyocyte-specific knockout of STRN Male STRN^fl/fl^/Cre^+/−^ mice (8 wks) were treated with corn-oil vehicle (COV) or tamoxifen (Tx; 40 mg/kg) and minipumps implanted to deliver acidified PBS (AcPBS) or 0.8 mg/kg/d AngII for 7 days. (**A**) B-mode images of the aorta at cardiac systole or diastole (upper and lower panels, respectively; images from each condition are from the same mouse). Aortic width measured after the aortic valve at its largest (i.e., immediately following cardiac contraction) and smallest diameter (with cardiac relaxation) as shown by the yellow lines. (**B**) Analysis of aortic diameter at cardiac systole and diastole plus the ratio between the two values. Individual datapoints are plotted with means ± SEM. Statistical analysis used two-way ANOVA with Holm-Sidak's post-test.

Finally, to determine if there were likely to be secondary consequences of the compromised cardiac response to AngII in mice with cardiomyocyte STRN knockout, we assessed the responsiveness of the aorta, focusing on the Windkessel effect [45]. This is seen in the large elastic arteries that distend when blood pressure increases with cardiac contraction in systole and recoil as blood pressure falls during diastole. The Windkessel effect is a system that dampens the large variation in blood pressure during the cardiac cycle and is lost with arterial stiffening resulting from aging or atherosclerosis [46]. We measured the width of the ascending aorta (immediately after the aortic valve and before the aortic arch) after systolic contraction when the aortic diameter is at its largest, and the narrowest diameter following cardiac relaxation. At 7 d, AngII had no significant effect on either measurement in mice treated with corn-oil vehicle alone. However, with cardiomyocyte deletion of STRN, the width of the aorta following relaxation (i.e., the smallest diameter) was significantly greater in mice treated with AngII than the aortas from the vehicle treated mice (Figure 9). This resulted in a significant decrease in the width ratio, suggesting that the loss of striatin in the heart had a secondary effect on the aorta with loss of compliance and reduction in the Windkessel effect.

## Discussion

Eukaryotic cellular responses are regulated by vast numbers of protein phosphorylation reactions, catalysed by over 500 different protein kinases in the mammalian kinome [47,48] and countered by a range of protein phosphatases [49]. We have detailed knowledge of how some key signalling pathways operate, but the regulation and roles of many protein kinases remain to be unravelled. Here, we focused on a relatively uninvestigated system, the STRIPAK complexes with a striatin isoform at the core, bringing together the most abundant protein phosphatase in the cell (PP2A) with key protein kinases (e.g., GCKs) to regulate their activation [25,26]. Our data indicate that the three striatin isoforms are all dysregulated in human failing hearts, but our studies with genetically altered mice place a particular emphasis on STRN itself in the development of cardiac hypertrophy induced by AngII treatment and, therefore, in the broader context of hypertensive heart disease.

**Figure 10:**
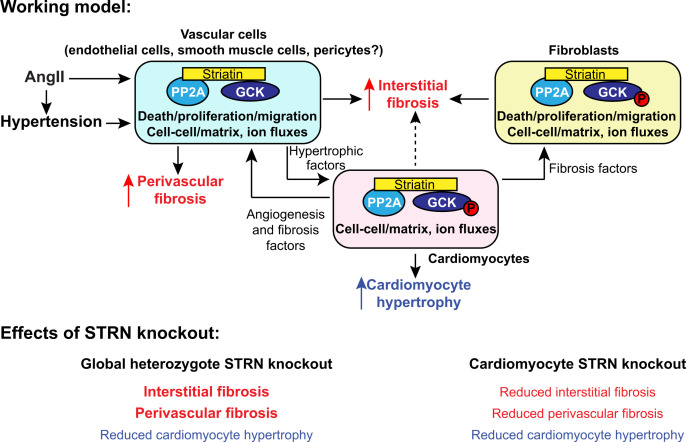
Working model of striatin signalling in cardiac hypertrophy induced by angiotensin II and conclusions from this study Previous studies place striatin at the core of STRIPAK complexes in which PP2A is brought into the vicinity of protein kinases (particularly those of GCK family). Here, PP2A dephosphorylates and inactivates GCKs. STRIPAKs are targeted to subcellular domains where they modulate cell–cell and cell–matrix interactions, regulate cell death or influence cell growth, proliferation and migration. In our working model, in addition to global effects of AngII to increase blood pressure, there are also local effects of AngII on cardiac cells. The increase in blood pressure, along with AngII itself have a primary effect on endothelial cells that are in direct contact with the blood and highly responsive to this hormone. In arterioles, there is additional interaction of AngII with smooth muscle cells that are also directly affected by blood pressure. These cells release pro-hypertrophic factors to promote cardiomyocyte hypertrophy. Cardiomyocytes release additional pro-fibrotic and pro-proliferative factors that affect other cardiac cells. The response involves STRN-based STRIPAKs in one or more of the cardiac cell types with, potentially, protein kinases such as GCKs being activated by reduced PP2A activity in the STRIPAK complex. The data from this study show that cardiomyocyte-specific knockout of STRN in mice reduced cardiomyocyte hypertrophy and cardiac fibrosis resulting from AngII treatment. AngII-induced cardiomyocyte hypertrophy was also inhibited in heterozygous global STRN knockout mice compared with wild-type littermates, but there was no effect on interstitial or perivascular fibrosis.

Our study was conducted in a context of the working model shown in Figure 10 in which STRN-based STRIPAKs operate in all cardiac cells. In addition to global effects of AngII to increase blood pressure, there are local effects of AngII on cardiac cells. The primary effect of both is likely to be on the vascular cells within the heart that are in direct contact with or close proximity to the blood, and which are highly responsive to this hormone (i.e., endothelial cells, smooth muscle cells and/or pericytes). Amongst other effects, AngII stimulates release of pro-hypertrophic factors (e.g., endothelin-1, Edn1 [50]) that act on cardiomyocytes, inducing cardiomyocyte hypertrophy. In turn, cardiomyocytes release factors that promote fibrosis (e.g., fibroblast growth factor 2, FGF2) and proliferation (e.g., EGF family ligands) in other cardiac cells. Our previous studies with BRAF knockout mice support this concept since *Edn1* and *FGF2* mRNAs are up-regulated in mouse hearts by AngII, but manipulation of cardiomyocyte signalling (with cardiomyocyte knockout of BRAF) selectively inhibits the increase in *FGF2* [34]. We propose that the response involves striatin-based STRIPAKs with activation of GCKs in one or more of the cardiac cell types.

Cardiomyocyte-specific knockout of STRN (with potential dysregulation and mislocalisation of one or more GCK) was sufficient to reduce cardiomyocyte hypertrophy (Figures 6-8). Pro-fibrotic cardiomyocyte-derived factors were also reduced, resulting in inhibition of fibrotic genes and overall reduction in cardiac fibrosis. Thus, striatin-based STRIPAKs are required for cardiomyocyte and cardiac hypertrophy in AngII-induced hypertension. With heterozygous global STRN knockout, there was no significant effect on interstitial or perivascular fibrosis induced by AngII compared with wild-type littermates, but there was greater suppression of cardiomyocyte hypertrophy (Figures 2-4). This model is more complicated, with reduction of STRN-based STRIPAKs in all cardiac cells, potentially disrupting the entire local cardiac response to AngII. Furthermore, loss of STRN in vascular smooth muscle and endothelial cells in peripheral resistance blood vessels carries a potential to affect blood pressure as discussed below. The loss of cardiomyocyte hypertrophy could be a combination of reduced hypertrophic signals from other cells and reduced cardiomyocyte hypertrophy signalling. The apparent lack of effect on fibrosis could be due to changes in pro- *vs* anti-fibrotic factors that were counterbalanced. However, we can only speculate and further research is needed on the role(s) of STRN in cardiac non-myocytes.

Previous studies in mice with global heterozygous STRN knockout used the same knockout-first system as we used here. The earlier studies developed from prior work demonstrating up-regulation of STRN in mouse heart and aorta by aldosterone, modulation of dietary salt or a combination of L-NAME (to inhibit NO production) and AngII [51]. STRN^+/−^ mice and their wild-type littermates have similar blood pressure when fed a restricted salt or normal diet (0.03% or 0.3–0.5% NaCl, respectively), but have an exaggerated increase in blood pressure on a higher salt diet (1.6% NaCl) along with enhanced contraction of aortic rings and reduced relaxation [3–5]. STRN^+/−^ mice also have an enhanced response to aldosterone with increased renal (though not cardiac) damage [5].

Interestingly, there is no significant difference in the renal or cardiac responses of STRN^+/−^ and wild-type mice in a hypertension model using L-NAME and AngII, despite an increase in blood pressure. This apparently contradicts our data, but L-NAME inhibits NO production and compromises endothelial cell function and vessel relaxation [52]. In Garza et al. [4], mice were treated with L-NAME for 7 d, before implantation of minipumps for delivery of 0.7 mg/kg/d AngII for 3 d in a regime that increased blood pressure. We used a similar dose of AngII over 7 d without L-NAME, a regime with a minimal effect on blood pressure over this time [39–41] and, arguably, a milder model. It remains possible that the blood pressure response to AngII alone (not measured in this study) could be affected in STRN^+/−^ mice if STRN-based STRIPAKs are involved in AngII receptor signalling in vascular smooth muscle and/or endothelial cells in peripheral resistance blood vessels. Deletion of type 1 AngII receptors (AT_1_Rs) in vascular smooth muscle cells in resistance vessels in mice reduces baseline blood pressure and inhibits the increase in blood pressure induced by AngII by 30% [53,54]. Thus, although there is no baseline difference between STRN^+/−^ mice and their wild-type littermates [3–5], the effect of AngII on blood pressure in heterozygote STRN^+/−^ mice could be compromised to some degree, possibly contributing to some of the effects on the heart that we detected (Figures 2-4). However, AngII increases NO production in endothelial cells [55], and expression of constitutively-active AT_1_Rs in endothelial cells decreases basal blood pressure with increased production of NO [56], potentially countering the effects in vascular smooth muscle cells. Further research is clearly important to assess the specific roles of STRN-based STRIPAKs in vascular smooth muscle cells and endothelial cells not only in the heart but also in peripheral blood vessels and the effects on blood pressure, alongside tissue pathologies.

Our studies assessed the effects of STRN deletion only in young male mice and in a context of AngII-induced hypertension using a dose of 0.8 mg/kg/d. The dosage was selected carefully because our pilot studies with a higher dose (0.9 mg/kg/d) resulted in increased mortality (20–30% over 3–7 d) due to rupture of a major blood vessel (unpublished data). The concentration of AngII we used is recognised as a slow pressor dose [39–41] and induced features of hypertensive heart disease in all three genetic models with cardiomyocyte hypertrophy and cardiac fibrosis even over 7 days. We have used the same dose in previous experiments including an assessment of the effects of dabrafenib (a BRAF inhibitor) on AngII-induced hypertension, in which we also demonstrated how the disease progresses towards heart failure over 28 d [57]. Further studies of STRN over this longer period may be useful to determine whether it plays a significant role in later stages of the disease. We have yet to assess the role of STRN or STRN3 in female mice. Here, it is necessary to consider that hypertension develops in males at an earlier age than females, and females have some protection against hypertension until after the menopause [58]. Thus, young female mice are resistant to AngII-induced hypertension and induction of menopause results in loss of this resistance [42,43]. Notably, protection is restored if mice continue to receive oestrogen. Overall, although there is emphasis on assessing and comparing males and females in all studies, particularly for hypertension, it seems more appropriate and necessary to consider the sexes separately allowing for menopausal status in females.

STRN3 was first identified as a nuclear antigen (S/G2 nuclear antigen, SG2NA) subject to cell cycle regulation [59] and, consequently, there is greater emphasis on its role in proliferating cells and cancer (e.g., [60]). However, it is expressed at significant levels in the heart and, of the multiple splice-variants, the dominant isoform is reported to be 78 kD, lacking two exons from the full-length 87 kDa variant [61,62]. Although other variants are reported in the heart, we detected a single dominant band above the 75 kDa marker, presumably corresponding to the 78 kDa STRN3 isoform (Supplementary Figures S4–S7). We are not aware of other published studies of STRN3 in the heart or of *in vivo* studies in STRN3 knockout mice. STRN3 was expressed in mouse and human hearts and was significantly upregulated in human failing hearts (Figure 1A–C). However, we did not detect any significant differences in cardiac function or dimensions between STRN3^+/−^ mice and their wild-type littermates, either at baseline or in response to AngII (Supplementary Figures S2,3, and Supplementary Tables S4, S6 and S7). The studies of STRN3^+/−^ and STRN^+/−^ mice were done in parallel and the negative results with STRN3^+/−^ mice emphasize the potential importance of STRN in the early adaptive response to AngII. However, STRN3 may play an important role in later phases of hypertension-induced cardiac dysfunction and/or in other cardiac pathologies (e.g., myocardial infarction that results in acute injury). It also has to be considered that we studied mice with heterozygote rather than homozygote gene deletion and study of homozygotes (not possible because of embryonic lethality) may have been more revealing. Because of this, it was important to adopt a conditional deletion approach to avoid problems during development.

Immunostaining studies place STRN at the intercalated disc in the heart suggesting it may regulate ion fluxes [6,30]. Consistent with this concept, reduced expression of STRN in boxer dogs is associated with ARVC and heart failure [6,7], both of which are associated with a higher risk of life-threatening ventricular arrhythmia and poor prognosis [63]. In addition, studies in cultured cardiomyocytes indicate that overexpression of striatin enhances contraction and STRN knockdown reduces contractility [64]. Human genome-wide association studies (GWAS) link the STRN gene with QRS/PR interval [10,11], further suggesting a role in regulating ion fluxes and contractility in cardiomyocytes. We did not detect any differences in cardiac function between STRN^+/−^ mice and their wild-type littermates, but it is unlikely that we would have detected arrhythmias with echocardiography in the relatively young mice we studied with a relatively low level of stress resulting from the dose and duration of AngII treatment. Greater differences would perhaps have been detected in older mice or with a more severe or prolonged stress (e.g., in a myocardial infarction model, prolonged treatment with AngII or with transverse aortic constriction).

Even though we saw no effect of heterozygous STRN knockout on cardiac function using echocardiography, the overall hypertrophic response induced by AngII was inhibited (Figure 2). This was due to a reduction in cardiomyocyte hypertrophy rather than fibrosis (Figures 3 and 4), suggesting that the phenotype resulted from decreased STRN expression in cardiomyocytes. To address this, we developed a model for homozygous cardiomyocyte-specific STRN knockout adopting a system for inducible and conditional gene deletion. This used a well-established approach using a tamoxifen-inducible Cre enzyme under the regulation of the MYH6 promoter [36], a system which avoids problems associated with development but raises additional concerns of potential cardiotoxicity from the Cre enzyme. This was minimised by only using mice hemizygous for Cre and by restraining temporal activation of the enzyme with just a single dose of tamoxifen to induce recombination. In hemizygous Cre^+/−^ mice, we detect no cardiotoxicity with or without AngII for at least the duration of the experiments reported here [33,35]. Others have used a similar approach and also report little cardiotoxicity [65]. Given the results with STRN^+/−^ mice, we anticipated that the hypertrophic response induced by AngII would be compromised by cardiomyocyte STRN knockout, and the increase in predicted LV mass estimated on echocardiograms was, indeed, reduced (Figure 6C). However, the degree of inhibition of cardiomyocyte hypertrophy appeared less than with the STRN^+/−^ mice and, in contrast to the STRN^+/−^ mice, there was substantial reduction in fibrosis (Figures 7 and 8). The mice were derived from sperm from our STRN^+/−^ colony so the difference is unlikely to be due to the genetic background. Thus, the effect on fibrosis is most probably a true reflection of the knockout system.

SNPs in the STRN gene have been linked to regulation of blood pressure and the development of heart failure using GWAS, but there are some difficulties with interpretation. The first SNP to be linked to QRS interval (rs17020136 [66]) was originally placed in the STRN gene, but is now linked to the adjacent HEATR5B gene in the EMBL-EBI GWAS Catalog (https://www.ebi.ac.uk/gwas), along with others associated with increased systolic blood pressure (rs146074994, 13408514 [9,67]). HEATR5B (HEAT repeat containing 5B) is a ubiquitously expressed protein-coding gene of unknown function and further studies of its role in blood pressure regulation may be useful. Nevertheless, SNPs in the STRN gene are also linked to increased blood pressure (rs2540923 [3], rs3770770 [9]) in addition to QRS/PR interval (rs3770770 [10], rs17496249 [11]), hypertrophic cardiomyopathy and heart failure (rs2003585 [12]). Many of the identified STRN SNPs associated with cardiac dysfunction are intronic, so the functional consequences are not clear. Nevertheless, linkage of the STRN gene with blood pressure along with studies in STRN^+/−^ mice have led to a clinical trial for use of mineralocorticoid receptor antagonists in hypertensive patients carrying STRN risk alleles [68].

Our data implicate cardiomyocyte STRN in cardiac hypertrophy, but provide limited insight into the mechanism of action. Striatin itself becomes hyperphosphorylated on inhibition of PP2A in cardiomyocytes [69], a modification which may modify subcellular localisation and/or binding partners. It may also be subject to ADP-ribosylation [70], although this has not been studied in the heart. The protein kinases identified in STRIPAK complex signalling belong to the GCK family with the GCKII (MST1 and MST2 [71]), GCKIII (MST3, MST4, YSK [60,72–74]), GCKV (SLIK in Drosophila; SLK and LOK are mammalian homologues [73,75]) and GCKVI (MAP4K4, TNIK, MINK1 [76–78]) subfamilies being specifically implicated to date. MST1/2, MST3, SLK and MAP4K4 are relatively highly expressed in adult rat cardiomyocytes [79], so these are the candidate kinases for cardiac adaptation to AngII. MST1/MST2 are involved in HIPPO signalling and regulation of cell survival/cell death in the heart [80]. Since cardiomyocyte MST1 knockout increases autophagic flux to alleviate AngII-induced cardiac damage [81], dysregulation of MST1 as a result of cardiomyocyte STRN knockout could have a similar effect and reduce cardiomyocyte hypertrophy. MAP4K4 associates with striatins in cardiomyocytes and is linked to human heart failure [69,82,83], so could also be involved. MST3 plays an important role in cell migration and is regulated acutely by phosphatase activity in cardiomyocytes [84,85], but there is little/no information on the role of SLK in heart. Whilst all of the kinases may interact with each of the striatins in experiments conducted *in vitro* or using overexpression approaches, specificity in terms of STRIPAK binding partners or subcellular targeting remains to be determined.

We tried to gain insight into which kinases may be involved in our study and whether global knockdown of STRN or STRN3, or cardiomyocyte STRN knockout had any effect on GCKs by immunoblotting mouse heart extracts (data not shown). Although the antibodies were adequate for studies of MST3 and MAP4K4 in rat cardiomyocytes [69,84], we failed to obtain reliable signals for these kinases in mouse hearts. Of a range of other antibodies, only MST1 produced a band of an appropriate molecular weight, but the results were variable and the reliability of the data is questionable. Even if we could detect these proteins, it would have been difficult to interpret the data because of the interconnecting networks of STRIPAK complexes. Clearly further studies are required to determine the nature of specific STRIPAK complexes in cardiomyocytes and the heart, along with their subcellular localisation, but *in vivo* gene deletion studies are probably not the best approach. For this stage of the research, it may be more appropriate to increase knowledge and understanding of the biochemical basis of the signalling pathway before trying to understand the implications for heart disease. These studies may benefit from the use of genetically modified systems for direct labelling of near-neighbour proteins that can then be identified and tracked. This technology is becoming available (e.g., Bio-ID [86]) and will be invaluable for understanding multiprotein systems such as those involved in STRIPAK signalling.

This study only considers the role of STRN and STRN3 in the early stages of cardiac remodelling induced by AngII, not the later stages associated with heart failure and decreased ejection fraction. Extending the study over a more prolonged period would enable further assessment of whether STRN knockdown or cardiomyocyte deletion could prevent this deterioration of cardiac function. Longer term studies would also help to determine if STRN3 plays an important role in developing heart failure, as suggested by the minor abnormalities in longitudinal strain we detected in STRN3^+/−^ mice treated with AngII over 7 d. We also did not consider the possible effects of STRN on arrhythmias and sudden death. We noted that the STRN mice under investigation in this study appeared more prone to sudden death than other genetically altered mice (e.g., those associated with BRAF [33,35]) we studied in parallel. However, there was no correlation with STRN expression (Supplementary Table S1), suggesting it was either coincidental or related to the background strain. Given the link between STRN and ARVC in boxer dogs [6,7] and SNPs in the STRN gene to hypertrophic cardiomyopathy, it will be important to conduct additional studies to assess possible arrhythmias in mice with STRN knockdown. Probably the greatest limitation of this study is the lack of knowledge of the STRIPAK complexes themselves. Thus, although the data suggest that inhibiting STRN will reduce cardiac hypertrophy induced by AngII, STRN potentially acts at the core of multiple complexes that regulate different GCKs, and one or more of these GCKs may be involved. Knowing which GCKs are involved and how they are regulated will be a crucial element for identifying specific targets for therapeutic manipulation of STRIPAK signalling.

In conclusion, our data indicate that STRN, but probably not STRN3, plays an important role in the early remodelling processes induced in the heart to AngII. There is clearly much research on STRN still to be done to understand its role in hypertensive heart disease, not only for cardiac pathologies (e.g., to understand the role of STRN in cardiac non-myocytes) but also for the rest of the cardiovascular system (e.g., STRIPAK complex involvement in blood pressure regulation and the peripheral vasculature). In addition, STRN3 and STRN4 remain to be investigated, along with the involvement of individual GCKs in specific STRIPAK complexes in each of the aforementioned cells. Nevertheless, the data in this study clearly identify striatin-based STRIPAKs as a novel signalling paradigm in the development of pathological cardiac hypertrophy. Understanding this system may provide therapeutic options for modulating the responses and managing progression of hypertensive heart disease.

## Clinical perspectives

- **Background:** Striatins form the core of ***STR***iatin-***I***nteracting ***P***hosphatase ***A***nd ***K***inase (STRIPAK) complexes that regulate crucial cellular processes such as those associated with heart failure.
- **Summary:** The three striatins are expressed in human hearts, with up-regulation of STRN and STRN3 in failing hearts, whilst studies in mice indicate that STRN is required in cardiomyocytes for early remodelling of the hypertensive heart.
- **Potential significance of results to human health and disease:** STRN-based STRIPAKs represent a novel signalling paradigm in the development of pathological cardiac hypertrophy, and modulating this system may provide therapeutic options for managing the cardiac effects of hypertensive heart disease.

## Supplementary Material

Supplementary Figures S1-S7 and Tables S1-S9

## Data Availability

All primary data are available from the corresponding author upon reasonable request.

## Open Access

Open access for this article was enabled by the participation of University of Reading in an all-inclusive *Read & Publish* agreement with Portland Press and the Biochemical Society under a transformative agreement with JISC.

## Abbreviations

AcPBS: acidified PBS
AngII: angiotensin II
AT_1_R: type 1 AngII receptor
CON: control
COV: corn-oil vehicle
DCM: dilated cardiomyopathy
FGF2: fibroblast growth factor 2
GCK: Germinal Centre Kinase
GWAS: genome-wide association studies
HF: heart failure
LV: left ventricle
LVID: left ventricle internal diameter
LVWT: left ventricle wall thickness
STRIPAK: ***STR***iatin-***I***nteracting ***P***hosphatase ***A***nd ***K***inase
TBST: Tris buffered saline containing 0.1% Tween 20
Tx: tamoxifen
WT: wild-type

## References

[1] Savarese G., Becher P.M., Lund L.H., Seferovic P., Rosano G.M.C. and Coats A. (2023) Global burden of heart failure: a comprehensive and updated review of epidemiology. Cardiovasc. Res. 118, 3272–3287 10.1093/cvr/cvac013 35150240

[2] Mills K.T., Stefanescu A. and He J. (2020) The global epidemiology of hypertension. Nat. Rev. Nephrol. 16, 223–237 10.1038/s41581-019-0244-2 32024986 PMC7998524

[3] Garza A.E., Rariy C.M., Sun B., Williams J.S., Lasky-Su J., Baudrand R. et al. (2015) Variants in striatin gene are associated with salt-sensitive blood pressure in mice and humans. Hypertension 65, 211–217 10.1161/HYPERTENSIONAHA.114.04233 25368024 PMC4617687

[4] Garza A.E., Pojoga L.H., Moize B., Hafiz W.M., Opsasnick L.A., Siddiqui W.T. et al. (2015) Critical role of striatin in blood pressure and vascular responses to dietary sodium intake. Hypertension 66, 674–680 10.1161/HYPERTENSIONAHA.115.05600 26169051 PMC4537321

[5] Garza A.E., Trefts E., Katayama Rangel I.A., Brooks D., Baudrand R., Moize B. et al. (2020) Striatin heterozygous mice are more sensitive to aldosterone-induced injury. J. Endocrinol. 245, 439–450 10.1530/JOE-19-0562 32229698 PMC7219220

[6] Meurs K.M., Mauceli E., Lahmers S., Acland G.M., White S.N. and Lindblad-Toh K. (2010) Genome-wide association identifies a deletion in the 3' untranslated region of striatin in a canine model of arrhythmogenic right ventricular cardiomyopathy. Hum. Genet. 128, 315–324 10.1007/s00439-010-0855-y 20596727 PMC2962869

[7] Meurs K.M., Stern J.A., Sisson D.D., Kittleson M.D., Cunningham S.M., Ames M.K. et al. (2013) Association of dilated cardiomyopathy with the striatin mutation genotype in boxer dogs. J. Vet. Intern. Med. 27, 1437–1440 10.1111/jvim.12163 24033487

[8] Gupta T., Connors M., Tan J.W., Manosroi W., Ahmed N., Ting P.Y. et al. (2017) Striatin gene polymorphic variants are associated with salt sensitive blood pressure in normotensives and hypertensives. Am. J. Hypertens. 31, 124–131 10.1093/ajh/hpx146 28985281 PMC5861567

[9] Plotnikov D., Huang Y., Khawaja A.P., Foster P.J., Zhu Z., Guggenheim J.A. et al. (2022) High blood pressure and intraocular pressure: a Mendelian randomization study. Invest. Ophthalmol. Vis. Sci. 63, 29 10.1167/iovs.63.6.29 35762941 PMC9251815

[10] van der Harst P., van Setten J., Verweij N., Vogler G., Franke L., Maurano M.T. et al. (2016) Genetic loci influencing myocardial mass. J. Am. Coll. Cardiol. 68, 1435–1448, 52 10.1016/j.jacc.2016.07.729 27659466 PMC5478167

[11] Ntalla I., Weng L.C., Cartwright J.H., Hall A.W., Sveinbjornsson G., Tucker N.R. et al. (2020) Multi-ancestry GWAS of the electrocardiographic PR interval identifies 202 loci underlying cardiac conduction. Nat. Commun. 11, 2542 10.1038/s41467-020-15706-x 32439900 PMC7242331

[12] Harper A.R., Goel A., Grace C., Thomson K.L., Petersen S.E., Xu X. et al. (2021) Common genetic variants and modifiable risk factors underpin hypertrophic cardiomyopathy susceptibility and expressivity. Nat. Genet. 53, 135–142 10.1038/s41588-020-00764-0 33495597 PMC8240954

[13] Levin M.G., Tsao N.L., Singhal P., Liu C., Vy H.M.T., Paranjpe I. et al. (2022) Genome-wide association and multi-trait analyses characterize the common genetic architecture of heart failure. Nat. Commun. 13, 6914 10.1038/s41467-022-34216-6 36376295 PMC9663424

[14] Surendran P., Feofanova E.V., Lahrouchi N., Ntalla I., Karthikeyan S., Cook J. et al. (2020) Discovery of rare variants associated with blood pressure regulation through meta-analysis of 1.3 million individuals. Nat. Genet. 52, 1314–1332 10.1038/s41588-020-00713-x 33230300 PMC7610439

[15] Zhou P. and Pu W.T. (2016) Recounting cardiac cellular composition. Circ. Res. 118, 368–370 10.1161/CIRCRESAHA.116.308139 26846633 PMC4755297

[16] Dorn G.W. II, Robbins J. and Sugden P.H. (2003) Phenotyping hypertrophy: eschew obfuscation. Circ. Res. 92, 1171–1175 10.1161/01.RES.0000077012.11088.BC 12805233

[17] Sheng S.Y., Li J.M., Hu X.Y. and Wang Y. (2023) Regulated cell death pathways in cardiomyopathy. Acta Pharmacol. Sin. 44, 1521–1535 10.1038/s41401-023-01068-9 36914852 PMC10374591

[18] Gogiraju R., Bochenek M.L. and Schafer K. (2019) Angiogenic endothelial cell signaling in cardiac hypertrophy and heart failure. Front Cardiovasc. Med. 6, 20 10.3389/fcvm.2019.00020 30895179 PMC6415587

[19] Suthahar N., Meijers W.C., Sillje H.H.W. and de Boer R.A. (2017) From inflammation to fibrosis-molecular and cellular mechanisms of myocardial tissue remodelling and perspectives on differential treatment opportunities. Curr. Heart Fail Rep. 14, 235–250 10.1007/s11897-017-0343-y 28707261 PMC5527069

[20] Mishra S. and Kass D.A. (2021) Cellular and molecular pathobiology of heart failure with preserved ejection fraction. Nat. Rev. Cardiol. 18, 400–423 10.1038/s41569-020-00480-6 33432192 PMC8574228

[21] Kurose H. (2021) Cardiac fibrosis and fibroblasts. Cells 10, 1716 10.3390/cells10071716 34359886 PMC8306806

[22] Kovacic J.C., Dimmeler S., Harvey R.P., Finkel T., Aikawa E., Krenning G. et al. (2019) Endothelial to mesenchymal transition in cardiovascular disease: JACC State-of-the-Art Review. J. Am. Coll. Cardiol. 73, 190–209 10.1016/j.jacc.2018.09.089 30654892 PMC6865825

[23] Reynhout S. and Janssens V. (2019) Physiologic functions of PP2A: lessons from genetically modified mice. Biochim. Biophys. Acta Mol. Cell. Res. 1866, 31–50 10.1016/j.bbamcr.2018.07.010 30030003

[24] Delpire E. (2009) The mammalian family of sterile 20p-like protein kinases. Pflugers Archives 458, 953–967 10.1007/s00424-009-0674-y 19399514

[25] Hwang J. and Pallas D.C. (2014) STRIPAK complexes: structure, biological function, and involvement in human diseases. Int. J. Biochem. Cell Biol. 47, 118–148 10.1016/j.biocel.2013.11.021 24333164 PMC3927685

[26] Kuck U., Radchenko D. and Teichert I. (2019) STRIPAK, a highly conserved signaling complex, controls multiple eukaryotic cellular and developmental processes and is linked with human diseases. Biol. Chem. 400, 1005–1022 10.1515/hsz-2019-0173 31042639

[27] Goudreault M., D'Ambrosio L.M., Kean M.J., Mullin M.J., Larsen B.G., Sanchez A. et al. (2009) A PP2A phosphatase high density interaction network identifies a novel striatin-interacting phosphatase and kinase complex linked to the cerebral cavernous malformation 3 (CCM3) protein. Mol. Cell. Proteomics 8, 157–171 10.1074/mcp.M800266-MCP200 18782753 PMC2621004

[28] Herzog F., Kahraman A., Boehringer D., Mak R., Bracher A., Walzthoeni T. et al. (2012) Structural probing of a protein phosphatase 2A network by chemical cross-linking and mass spectrometry. Science 337, 1348–1352 10.1126/science.1221483 22984071

[29] Couzens A.L., Knight J.D., Kean M.J., Teo G., Weiss A., Dunham W.H. et al. (2013) Protein interaction network of the mammalian Hippo pathway reveals mechanisms of kinase-phosphatase interactions. Sci. Signal. 6, rs15 10.1126/scisignal.2004712 24255178

[30] Franke W.W., Rickelt S., Zimbelmann R., Dorflinger Y., Kuhn C., Frey N. et al. (2014) Striatins as plaque molecules of zonulae adhaerentes in simple epithelia, of tessellate junctions in stratified epithelia, of cardiac composite junctions and of various size classes of lateral adherens junctions in cultures of epithelia- and carcinoma-derived cells. Cell Tissue Res. 357, 645–665 10.1007/s00441-014-2053-z 25501894 PMC4341017

[31] Heinig M., Adriaens M.E., Schafer S., van Deutekom H.W.M., Lodder E.M., Ware J.S. et al. (2017) Natural genetic variation of the cardiac transcriptome in non-diseased donors and patients with dilated cardiomyopathy. Genome Biol. 18, 170 10.1186/s13059-017-1286-z 28903782 PMC5598015

[32] Love M.I., Huber W. and Anders S. (2014) Moderated estimation of fold change and dispersion for RNA-seq data with DESeq2. Genome Biol. 15, 550 10.1186/s13059-014-0550-8 25516281 PMC4302049

[33] Clerk A., Meijles D.N., Hardyman M.A., Fuller S.J., Chothani S.P., Cull J.J. et al. (2022) Cardiomyocyte BRAF and type 1 RAF inhibitors promote cardiomyocyte and cardiac hypertrophy in mice in vivo. Biochem. J. 479, 401–424 10.1042/BCJ20210615 35147166 PMC8883496

[34] Marshall J.J., Cull J.J., Alharbi H.O., Zaw Thin M., Cooper S.T., Barrington C. et al. (2022) PKN2 deficiency leads both to prenatal congenital cardiomyopathy and defective angiotensin II stress responses. Biochem. J. 479, 1467–1486 10.1042/BCJ20220281 35730579 PMC9342899

[35] Alharbi H.O., Hardyman M.A., Cull J.J., Markou T., Cooper S.T.E., Glennon P.E. et al. (2022) Cardiomyocyte BRAF is a key signalling intermediate in cardiac hypertrophy in mice. Clin. Sci. (Lond.) 136, 1661–1681 10.1042/CS20220607 36331065 PMC9679367

[36] Sohal D.S., Nghiem M., Crackower M.A., Witt S.A., Kimball T.R., Tymitz K.M. et al. (2001) Temporally regulated and tissue-specific gene manipulations in the adult and embryonic heart using a tamoxifen-inducible Cre protein. Circ. Res. 89, 20–25 10.1161/hh1301.092687 11440973

[37] Meijles D.N., Cull J.J., Markou T., Cooper S.T.E., Haines Z.H.R., Fuller S.J. et al. (2020) Redox regulation of cardiac ASK1 (Apoptosis Signal-Regulating Kinase 1) controls p38-MAPK (mitogen-activated protein kinase) and orchestrates cardiac remodeling to hypertension. Hypertension 76, 1208–1218 10.1161/HYPERTENSIONAHA.119.14556 32903101 PMC7480944

[38] Marshall A.K., Barrett O.P.T., Cullingford T.E., Shanmugasundram A., Sugden P.H. and Clerk A. (2010) ERK1/2 signaling dominates over RhoA signaling in regulating early changes in RNA expression induced by endothelin-1 in neonatal rat cardiomyocytes. PLoS ONE 5, e10027 10.1371/journal.pone.0010027 20368814 PMC2848868

[39] Zimmerman M.C., Lazartigues E., Sharma R.V. and Davisson R.L. (2004) Hypertension caused by angiotensin II infusion involves increased superoxide production in the central nervous system. Circ. Res. 95, 210–216 10.1161/01.RES.0000135483.12297.e4 15192025

[40] Patel J., Douglas G., Kerr A.G., Hale A.B. and Channon K.M. (2018) Effect of irradiation and bone marrow transplantation on angiotensin II-induced aortic inflammation in ApoE knockout mice. Atherosclerosis 276, 74–82 10.1016/j.atherosclerosis.2018.07.019 30048944 PMC6143484

[41] Capone C., Faraco G., Peterson J.R., Coleman C., Anrather J., Milner T.A. et al. (2012) Central cardiovascular circuits contribute to the neurovascular dysfunction in angiotensin II hypertension. J. Neurosci. 32, 4878–4886 10.1523/JNEUROSCI.6262-11.2012 22492044 PMC3328774

[42] Pollow D.P. Jr, Romero-Aleshire M.J., Sanchez J.N., Konhilas J.P. and Brooks H.L. (2015) ANG II-induced hypertension in the VCD mouse model of menopause is prevented by estrogen replacement during perimenopause. Am. J. Physiol. Regul. Integr. Comp. Physiol. 309, R1546–R1552 10.1152/ajpregu.00170.2015 26491098 PMC4698416

[43] Brooks H.L., Pollow D.P. and Hoyer P.B. (2016) The VCD mouse model of menopause and perimenopause for the study of sex differences in cardiovascular disease and the metabolic syndrome. Physiology (Bethesda). 31, 250–257 10.1152/physiol.00057.2014 27252160 PMC5504385

[44] Jahn H.M., Kasakow C.V., Helfer A., Michely J., Verkhratsky A., Maurer H.H. et al. (2018) Refined protocols of tamoxifen injection for inducible DNA recombination in mouse astroglia. Sci. Rep. 8, 5913 10.1038/s41598-018-24085-9 29651133 PMC5897555

[45] Belz G.G. (1995) Elastic properties and Windkessel function of the human aorta. Cardiovasc. Drugs Ther. 9, 73–83 10.1007/BF00877747 7786838

[46] Pierce G.L., Coutinho T.A., DuBose L.E. and Donato A.J. (2022) Is it good to have a stiff aorta with aging? Causes and consequences. Physiology (Bethesda). 37, 154–173 10.1152/physiol.00035.2021 34779281 PMC8977146

[47] Manning G., Whyte D.B., Martinez R., Hunter T. and Sudarsanam S. (2002) The protein kinase complement of the human genome. Science 298, 1912–1934 10.1126/science.1075762 12471243

[48] Caenepeel S., Charydczak G., Sudarsanam S., Hunter T. and Manning G. (2004) The mouse kinome: discovery and comparative genomics of all mouse protein kinases. Proc. Natl. Acad. Sci. U.S.A. 101, 11707–11712 10.1073/pnas.0306880101 15289607 PMC511041

[49] Nguyen H. and Kettenbach A.N. (2023) Substrate and phosphorylation site selection by phosphoprotein phosphatases. Trends Biochem. Sci. 48, 713–725 10.1016/j.tibs.2023.04.004 37173206 PMC10523993

[50] Marasciulo F.L., Montagnani M. and Potenza M.A. (2006) Endothelin-1: the yin and yang on vascular function. Curr. Med. Chem. 13, 1655–1665 10.2174/092986706777441968 16787211

[51] Pojoga L.H., Coutinho P., Rivera A., Yao T.M., Maldonado E.R., Youte R. et al. (2012) Activation of the mineralocorticoid receptor increases striatin levels. Am. J. Hypertens. 25, 243–249 10.1038/ajh.2011.197 22089104 PMC3773217

[52] Evora P.R., Evora P.M., Celotto A.C., Rodrigues A.J. and Joviliano E.E. (2012) Cardiovascular therapeutics targets on the NO-sGC-cGMP signaling pathway: a critical overview. Curr. Drug Targets 13, 1207–1214 10.2174/138945012802002348 22716077

[53] Sparks M.A., Stegbauer J., Chen D., Gomez J.A., Griffiths R.C., Azad H.A. et al. (2015) Vascular type 1A angiotensin II receptors control BP by regulating renal blood flow and urinary sodium excretion. J. Am. Soc. Nephrol. 26, 2953–2962 10.1681/ASN.2014080816 25855778 PMC4657834

[54] Rianto F., Hoang T., Revoori R. and Sparks M.A. (2021) Angiotensin receptors in the kidney and vasculature in hypertension and kidney disease. Mol. Cell. Endocrinol. 529, 111259 10.1016/j.mce.2021.111259 33781840

[55] Yan C., Kim D., Aizawa T. and Berk B.C. (2003) Functional interplay between angiotensin II and nitric oxide: cyclic GMP as a key mediator. Arterioscler. Thromb. Vasc. Biol. 23, 26–36 10.1161/01.ATV.0000046231.17365.9D 12524221

[56] Ramchandran R., Takezako T., Saad Y., Stull L., Fink B., Yamada H. et al. (2006) Angiotensinergic stimulation of vascular endothelium in mice causes hypotension, bradycardia, and attenuated angiotensin response. Proc. Natl. Acad. Sci. U.S.A. 103, 19087–19092 10.1073/pnas.0602715103 17148616 PMC1748181

[57] Meijles D.N., Cull J.J., Cooper S.T.E., Markou T., Hardyman M.A., Fuller S.J. et al. (2021) The anti-cancer drug dabrafenib is not cardiotoxic and inhibits cardiac remodelling and fibrosis in a murine model of hypertension. Clin. Sci. (Lond.) 135, 1631–1647 10.1042/CS20210192 34296750 PMC8302807

[58] Pitha J., Vaneckova I. and Zicha J. (2023) Hypertension after the menopause: what can we learn from experimental studies? Physiol. Res. 72, S91–S112 10.33549/physiolres.935151 37565415 PMC10660576

[59] Muro Y., Chan E.K., Landberg G. and Tan E.M. (1995) A cell-cycle nuclear autoantigen containing WD-40 motifs expressed mainly in S and G2 phase cells. Biochem. Biophys. Res. Commun. 207, 1029–1037 10.1006/bbrc.1995.1288 7864889

[60] Madsen C.D., Hooper S., Tozluoglu M., Bruckbauer A., Fletcher G., Erler J.T. et al. (2015) STRIPAK components determine mode of cancer cell migration and metastasis. Nat. Cell Biol. 17, 68–80 10.1038/ncb3083 25531779 PMC5354264

[61] Jain B.P., Chauhan P., Tanti G.K., Singarapu N., Ghaskadbi S. and Goswami S.K. (2015) Tissue specific expression of SG2NA is regulated by differential splicing, RNA editing and differential polyadenylation. Gene 556, 119–126 10.1016/j.gene.2014.11.045 25459749

[62] Sanghamitra M., Talukder I., Singarapu N., Sindhu K.V., Kateriya S. and Goswami S.K. (2008) WD-40 repeat protein SG2NA has multiple splice variants with tissue restricted and growth responsive properties. Gene 420, 48–56 10.1016/j.gene.2008.04.016 18571342

[63] Krahn A.D., Wilde A.A.M., Calkins H., La Gerche A., Cadrin-Tourigny J., Roberts J.D. et al. (2022) Arrhythmogenic right ventricular cardiomyopathy. JACC Clin. Electrophysiol. 8, 533–553 10.1016/j.jacep.2021.12.002 35450611

[64] Nader M., Alotaibi S., Alsolme E., Khalil B., Abu-Zaid A., Alsomali R. et al. (2017) Cardiac striatin interacts with caveolin-3 and calmodulin in a calcium sensitive manner and regulates cardiomyocyte spontaneous contraction rate. Can. J. Physiol. Pharmacol. 95, 1306–1312 10.1139/cjpp-2017-0155 28825318

[65] Hougen K., Aronsen J.M., Stokke M.K., Enger U., Nygard S., Andersson K.B. et al. (2010) Cre-loxP DNA recombination is possible with only minimal unspecific transcriptional changes and without cardiomyopathy in Tg(alphaMHC-MerCreMer) mice. Am. J. Physiol. Heart Circ. Physiol. 299, H1671–H1678 10.1152/ajpheart.01155.2009 20802136

[66] Sotoodehnia N., Isaacs A., de Bakker P.I., Dorr M., Newton-Cheh C., Nolte I.M. et al. (2010) Common variants in 22 loci are associated with QRS duration and cardiac ventricular conduction. Nat. Genet. 42, 1068–1076 10.1038/ng.716 21076409 PMC3338195

[67] Giri A., Hellwege J.N., Keaton J.M., Park J., Qiu C., Warren H.R. et al. (2019) Trans-ethnic association study of blood pressure determinants in over 750,000 individuals. Nat. Genet. 51, 51–62 10.1038/s41588-018-0303-9 30578418 PMC6365102

[68] Stone I.B., Green J., Koefoed A.W., Hornik E.S., Williams J.S., Adler G.K. et al. (2021) Striatin genotype-based, mineralocorticoid receptor antagonist-driven clinical trial: study rationale and design. Pharmacogenet Genomics 31, 83–88 10.1097/FPC.0000000000000425 33904521 PMC10352129

[69] Fuller S.J., Edmunds N.S., McGuffin L.J., Hardyman M.A., Cull J.J., Alharbi H.O. et al. (2021) MAP4K4 expression in cardiomyocytes: multiple isoforms, multiple phosphorylations and interactions with striatins. Biochem. J. 478, 2121–2143 10.1042/BCJ20210003 34032269 PMC8203206

[70] Guettler S., LaRose J., Petsalaki E., Gish G., Scotter A., Pawson T. et al. (2011) Structural basis and sequence rules for substrate recognition by Tankyrase explain the basis for cherubism disease. Cell 147, 1340–1354 10.1016/j.cell.2011.10.046 22153077

[71] Tang Y., Chen M., Zhou L., Ma J., Li Y., Zhang H. et al. (2019) Architecture, substructures, and dynamic assembly of STRIPAK complexes in Hippo signaling. Cell Discov. 5, 3 10.1038/s41421-018-0077-3 30622739 PMC6323126

[72] Gordon J., Hwang J., Carrier K.J., Jones C.A., Kern Q.L., Moreno C.S. et al. (2011) Protein phosphatase 2a (PP2A) binds within the oligomerization domain of striatin and regulates the phosphorylation and activation of the mammalian Ste20-Like kinase Mst3. BMC Biochem. 12, 54 10.1186/1471-2091-12-54 21985334 PMC3217859

[73] Kean M.J., Ceccarelli D.F., Goudreault M., Sanches M., Tate S., Larsen B. et al. (2011) Structure-function analysis of core STRIPAK proteins: a signaling complex implicated in Golgi polarization. J. Biol. Chem. 286, 25065–25075 10.1074/jbc.M110.214486 21561862 PMC3137080

[74] Ceccarelli D.F., Laister R.C., Mulligan V.K., Kean M.J., Goudreault M., Scott I.C. et al. (2011) CCM3/PDCD10 heterodimerizes with germinal center kinase III (GCKIII) proteins using a mechanism analogous to CCM3 homodimerization. J. Biol. Chem. 286, 25056–25064 10.1074/jbc.M110.213777 21561863 PMC3137079

[75] De Jamblinne C.V., Decelle B., Dehghani M., Joseph M., Sriskandarajah N., Leguay K. et al. (2020) STRIPAK regulates Slik localization to control mitotic morphogenesis and epithelial integrity. J. Cell Biol. 219, e201911035 10.1083/jcb.201911035 32960945 PMC7594492

[76] Hyodo T., Ito S., Hasegawa H., Asano E., Maeda M., Urano T. et al. (2012) Misshapen-like kinase 1 (MINK1) is a novel component of striatin-interacting phosphatase and kinase (STRIPAK) and is required for the completion of cytokinesis. J. Biol. Chem. 287, 25019–25029 10.1074/jbc.M112.372342 22665485 PMC3408143

[77] Kim J.W., Berrios C., Kim M., Schade A.E., Adelmant G., Yeerna H. et al. (2020) STRIPAK directs PP2A activity toward MAP4K4 to promote oncogenic transformation of human cells. Elife 9, e53003 10.7554/eLife.53003 31913126 PMC6984821

[78] Migliavacca J., Zullig B., Capdeville C., Grotzer M.A. and Baumgartner M. (2022) Cooperation of striatin 3 and MAP4K4 promotes growth and tissue invasion. Commun. Biol. 5, 795 10.1038/s42003-022-03708-y 35941177 PMC9360036

[79] Fuller S.J., Osborne S.A., Leonard S.J., Hardyman M.A., Vaniotis G., Allen B.G. et al. (2015) Cardiac protein kinases: the cardiomyocyte kinome and differential kinase expression in human failing hearts. Cardiovasc. Res. 108, 87–98 10.1093/cvr/cvv210 26260799

[80] Wang J., Liu S., Heallen T. and Martin J.F. (2018) The Hippo pathway in the heart: pivotal roles in development, disease, and regeneration. Nat. Rev. Cardiol. 15, 672–684 10.1038/s41569-018-0063-3 30111784

[81] Cheng Z., Zhang M., Hu J., Lin J., Feng X., Wang S. et al. (2018) Mst1 knockout enhances cardiomyocyte autophagic flux to alleviate angiotensin II-induced cardiac injury independent of angiotensin II receptors. J. Mol. Cell Cardiol. 125, 117–128 10.1016/j.yjmcc.2018.08.028 30193956

[82] Golforoush P.A., Narasimhan P., Chaves-Guerrero P.P., Lawrence E., Newton G., Yan R. et al. (2020) Selective protection of human cardiomyocytes from anthracycline cardiotoxicity by small molecule inhibitors of MAP4K4. Sci. Rep. 10, 12060 10.1038/s41598-020-68907-1 32694738 PMC7374628

[83] Fiedler L.R., Chapman K., Xie M., Maifoshie E., Jenkins M., Golforoush P.A. et al. (2019) MAP4K4 inhibition promotes survival of human stem cell-derived cardiomyocytes and reduces infarct size in vivo. Cell Stem Cell. 24, 579e512–591e512 10.1016/j.stem.2019.01.013 30853557 PMC6458995

[84] Fuller S.J., McGuffin L.J., Marshall A.K., Giraldo A., Pikkarainen S., Clerk A. et al. (2012) A novel non-canonical mechanism of regulation of MST3 (mammalian Sterile20-related kinase 3). Biochem. J. 442, 595–610 10.1042/BJ20112000 22229648 PMC3286863

[85] Sugden P.H., McGuffin L.J. and Clerk A. (2013) SOcK, MiSTs, MASK and STicKs: the germinal centre kinase III (GCKIII) kinases and their heterologous protein-protein interactions. Biochem. J. 454, 13–30 10.1042/BJ20130219 23889253

[86] Roux K.J., Kim D.I., Burke B. and May D.G. (2018) BioID: a screen for protein-protein interactions. Curr. Protoc. Protein Sci. 91, 19.23.11–19.23.15 10.1002/cpps.51 PMC6028010 29516480

