# Supplementary material for: Striatin plays a major role in angiotensin II-induced cardiomyocyte and cardiac hypertrophy in mice in vivo
Source: Clin Sci (Lond). 2024 May 22;138(10):573–97. doi: 10.1042/CS20240496 (PMC11130554; doi:10.1042/CS20240496)

Supplementary information:

STRN, but not STRN3, plays a major role in angiotensin II-induced cardiomyocyte and cardiac hypertrophy

Joshua J Cull[1], Susanna TE Cooper[2*], Hajed O Alharbi[1#], Sonia P Chothani[3], Owen JL Rackham[3,4], Daniel N Meijles[2], Philip R Dash[1], Risto Kerkelä[5], Neil Ruparelia[1,6], Peter H Sugden[1], Angela Clerk[1]

[1]School of Biological Sciences, University of Reading, Reading, UK.
[2]Molecular and Clinical Sciences Institute, St. George's University of London, London, UK.
[3]Program in Cardiovascular and Metabolic Disorders, Duke-National University of Singapore Medical School, Singapore.
[4]School of Biological Sciences, University of Southampton, Southampton, UK.
[5]Research Unit of Biomedicine and Internal Medicine, Medical Research Centre Oulu (Oulu University Hospital) and Biocenter Oulu, University of Oulu, Oulu, Finland.
[6]Department of Cardiology, Royal Berkshire Hospital, Reading, UK.

Supplementary Table S1. Mouse weights.

Supplementary Table S2. Primers for genotyping and confirmation of recombination.

Supplementary S3. qPCR primers.

Supplementary Table S4. Baseline echocardiography data for STRN$^{+/-}$ and STRN3$^{+/-}$ male mice and their wild-type (STRN$^{+/+}$ and STRN3$^{+/+}$) male littermates.

Supplementary Table S5. Echocardiography data for STRN$^{+/-}$ male mice and wild-type (STRN$^{+/+}$) male littermates treated with acidified PBS (AcPBS) or 0.8 mg/kg/d AngII for 3 or 7 d: M-mode data.

Supplementary Table S6. Echocardiography data for STRN3$^{+/-}$ male mice and wild-type (STRN3$^{+/+}$) male littermates treated with acidified PBS (AcPBS) or 0.8 mg/kg/d AngII for 3 or 7 d: M-mode data

Supplementary Table S7. Echocardiography data for STRN$^{+/-}$ and STRN3$^{+/-}$ male mice and wild-type (STRN$^{+/+}$ and STRN3$^{+/+}$) male littermates treated with acidified PBS (AcPBS) or 0.8 mg/kg/d AngII for 7 d: B-mode data and speckle tracking.

Supplementary Table S8. Echocardiography data for STRN3$^{fl/fl}$/Cre$^{+/-}$ mice: M-mode data.

Supplementary Table S9. Echocardiography data for STRN3$^{fl/fl}$/Cre$^{+/-}$ mice: B-mode data and speckle tracking.

Supplementary Figure S1. Global heterozygous STRN3 knockout in mice.

Supplementary Figure S2. Heterozygous global deletion of STRN does not affect the hypertrophic response to AngII.

Supplementary Figure S3. Heterozygous global deletion of STRN3 does not affect cardiomyocyte hypertrophy or cardiac fibrosis induced by AngII.

Supplementary Figure S4. Full images for immunoblots in Figure 1C.

Supplementary Figure S5. Full images for immunoblots in Figure 1F.

Supplementary Figure S6. Full images for immunoblots in Supplementary Figure S2C.

Supplementary Figure S7. Full images for immunoblots in Figure 5D.

Supplementary Table S1. Mouse weights. Male mice (8 weeks) were allocated to groups on a random basis. $STRN^{+/-}$ and $STRN3^{+/-}$ heterozygous knockout mice and their wild-type (WT) $STRN^{+/+}$ and $STRN3^{+/+}$ littermates were treated with acidified PBS (AcPBS) or 0.8 mg/kg/d angiotensin II (AngII). $STRN^{fl/fl}$/$Cre^{+/-}$ mice were treated with 40 mg/kg tamoxifen in corn-oil or with corn-oil vehicle (COV) with or without AcPBS or AngII. Weights (g) were taken at the start of the study with the first baseline echocardiogram (Start), immediately after minipump surgery, and when mice were culled (End). Weights post-surgery and at the end included the minipumps. N values are provided with exclusions due to mortality indicated in parentheses.

Study	Condition	Start		Post-minipump		End		
		Mean	SD	Mean	SD	Mean	SD	n
**STRN study group**								
$STRN^{+/+}$ (WT)	AcPBS	25.16	1.38	26.94	1.46	27.59	1.49	16
	AngII	24.77	1.57	26.46	1.42	26.65	1.38	15 (1)
$STRN^{+/-}$	AcPBS	25.00	0.92	26.76	0.95	27.52	0.78	10
	AngII	24.78	1.38	26.49	1.26	26.85	1.57	17
**STRN3 study group**								
$STRN3^{+/+}$ (WT)	AcPBS	23.62	2.11	25.44	1.71	26.07	1.66	16
	AngII	24.31	1.46	25.79	1.44	26.41	1.69	14
$STRN3^{+/-}$	AcPBS	23.26	2.20	24.85	2.01	25.76	2.15	11
	AngII	23.32	1.68	25.64	1.43	25.78	1.21	11
**$STRN^{fl/fl}$/$Cre^{+/-}$**	COV/AcPBS	22.21	1.53	24.58	2.06	25.27	1.76	10
	Tx/AcPBS	22.82	1.70	25.79	1.73	26.40	1.31	10
	COV/AngII	21.98	2.04	24.12	1.61	24.04	1.66	9 (2)
	Tx/AngII	25.38	1.70	25.38	1.70	25.48	1.86	8 (1)

Supplementary Table S2. Primers for genotyping and confirmation of recombination.

Mouse strain	Forward primer	Reverse primer	Size (bp)	Annealing temp.
**Genotyping**				
STRN knockout	GAGATGGCGCAACGCAATTAATG	GAAGTGCATGGGAAGTCAGTACACG	296	51°C
STRN3 knockout	GAGATGGCGCAACGCAATTAATG	ACCTGAGCCAAATTCACCCAAAACC	334	51°C
Cre^-	TCTATTGCACACAGCAATCCA	CCAACTCTTGTGAGAGGAGCA	305	52°C
Cre^+	TCTATTGCACACAGCAATCCA	CCAGCATTGTGAGAACAAGG	285	52°C
**Recombination**				
Post-FLP	TGAATTATTGGAGTTTTGTTTCAGACC	GCACAGACAGACCTTCATGCTAACC	630	53°C
Post-FLP Post-Cre	TGAATTATTGGAGTTTTGTTTCAGACC	GAAGTGCATGGGAAGTCAGTACACG	666	53°C

Supplementary S3. qPCR primers.

Gene Symbol	Accession No.	Sense Primer (5'→3')	Antisense Primer (5'→3')
Cdh1	NM_009864.3	GTCTCCTCATGGCTTTGC	CTTTAGATGCCGCTTCAC
Cdh5	NM_009868.4	TCTTGCCAGCAAACTCTCCT	TTGGAATCAAATGCACATCG
Col1a1	NM_007742	TCGTGGCTTCTCTGGTCTC	CCGTTGAGTCCGTCTTTGC
Col2a1	NM_00111351	GACGAGGCAGACAGTACCTTG	GATGCTCTCAATCTGGTTGTTCAG
Col3a1	NM_009930.2	GGAACCTGGTTTCTTCTCACC	TAGGACTGACCAAGGTGGCT
Col4a1	NM_009931.2	TGTGGGCCAGCCAGGCATTG	CAGGGGGTCCGATCGCTCCA
Ctgf	NM_010217	GCACACCGCACAGAACCA	ATGGCAGGCACAGGTCTTG
Ddr2	NM_022563.2	GCACTTGGTGAATTAATTAGAATCCTG	GGACAACTAAATGGTCCCTCCC
Fn1	NM_010233	AAGAGGACGTTGCAGAGCTA	AGACACTGGAGACACTGACTAA
Gapdh	NM_008084.2	TCACCACCATGGAGAAGGC	GCTAAGCAGTTGGTGGTGCA
Myh7	NM_080728	CATGCCAACCGTATGGCTG	GTTCCACGATGGCGATGTTC
Ng2 (Cspg4)	NM_139001.2	TTGGCTACGTGAAGATAGGG	AGCACGATGACTCTGAGACC
Nppa	NM_008725	GATGGATTTCAAGAACCTGCTAGA	CTTCCTCAGTCTGCTCACTCA
Nppb	NM_008726	TCCAGCAGAGACCTCAAAATTC	CAGTGCGTTACAGCCCAAA
Postn	NM_015784	TTCCTCTCCTGCCCTTATATGC	CCTGATCCCGACCCCTGAT
Tagln	NM_011526.5	GACTGCACTTCTCGGCTCAT	CCGAAGCTACTCTCCTTCCA
Timp1	NM_011593	TACGCCTACACCCCAGTCAT	GCCCGTGATGAGAAACTCTTC

Supplementary Table S4. Baseline echocardiography data for STRN$^{+/-}$ and STRN3$^{+/-}$ male mice and their wild-type (STRN$^{+/+}$ and STRN3$^{+/+}$) male littermates. Short axis M-mode images were analysed using VevoLab software. LV, left ventricle; ID, internal diameter; AW, anterior wall; PW, posterior wall; d, diastole; s, systole; wall thickness (WT) = AW+ PW; SV, stroke volume; CO, cardiac output; EF, ejection fraction; FS, fractional shortening. Long axis B-mode images were analysed using speckle-tracking software. EDV, End diastolic volume; ESV, End systolic volume; EDLVM, End diastolic left ventricle mass; ESLVM, End systolic left ventricle mass; GLS, global longitudinal strain; GCS, Global circumferential strain.

	**STRN$^{+/+}$ (n=31)**		**STRN$^{+/-}$ (n=26)**		**STRN3$^{+/+}$ (n=27)**		**STRN3$^{+/-}$ (n=19)**	
	**Mean**	**SD**	**Mean**	**SD**	**Mean**	**SD**	**Mean**	**SD**
**M-Mode**								
Heart Rate (bpm)	483	28	471	44	502	38	498	19
SV (µl)	42.16	8.83	42.31	6.27	40.68	4.94	41.36	7.78
CO (ml/min)	20.33	7.85	19.97	7.01	20.71	6.32	20.56	6.00
EF (%)	57.41	5.31	55.65	4.58	53.41	4.17	56.14	4.02
FS (%)	30.10	4.33	28.90	3.87	27.48	3.13	29.25	3.83
LVID; d (mm)	2.853	0.319	2.954	0.300	3.018	0.274	2.894	0.272
LVID; s (mm)	4.076	0.295	4.147	0.239	4.153	0.209	4.084	0.274
LVAW; d (mm)	1.043	0.099	1.039	0.094	1.072	0.079	1.078	0.077
LVAW; s (mm)	0.789	0.062	0.790	0.079	0.780	0.058	0.757	0.051
LVPW; d (mm)	0.999	0.116	0.991	0.093	0.986	0.081	1.007	0.083
LVPW; s (mm)	0.703	0.068	0.702	0.050	0.698	0.039	0.697	0.048
WT; d (mm)	2.042	0.201	2.030	0.150	2.059	0.148	2.085	0.141
WT; s (mm)	1.492	0.114	1.492	0.096	1.477	0.086	1.454	0.083
**B-mode**	**Mean**	**SD**	**Mean**	**SD**	**Mean**	**SD**	**Mean**	**SD**
Heart Rate (bpm)	484	31	473	54	480	40	472	40
SV (µl)	29.96	5.84	31.49	6.06	28.89	4.64	28.16	7.25
CO (ml/min)	14.46	2.85	14.99	3.82	13.87	2.47	13.13	2.71
EF (%)	57.54	6.86	58.14	6.45	56.26	8.10	57.38	7.83
FS (%)	30.73	5.68	30.71	4.96	28.34	6.38	30.24	5.82
EDV (µl)	52.07	9.17	54.17	9.96	51.57	8.40	48.86	9.88
ESV (µl)	22.12	5.61	22.68	5.88	22.68	6.52	20.70	5.43
EDLVM (mg)	53.90	5.43	54.21	5.83	53.24	5.20	50.75	5.79
ESLVM (mg)	56.03	5.76	56.11	6.20	55.99	5.94	52.99	6.01
GLS (%)	-20.27	2.91	-19.80	3.44	-20.76	4.97	-20.06	3.16
GCS (%)	-20.84	3.48	-20.83	3.40	-20.30	4.01	-19.98	3.34

Supplementary Table S5. Echocardiography data for STRN$^{+/-}$ male mice and wild-type (STRN$^{+/+}$) male littermates treated with acidified PBS (AcPBS) or 0.8 mg/kg/d AngII for 3 or 7 d: M-mode data. Short axis M-mode images were analysed using VevoLab software. LV, left ventricle; ID, internal diameter; AW, anterior wall; PW, posterior wall; d, diastole; s, systole; wall thickness (WT) = AW+ PW; SV, stroke volume; CO, cardiac output; EF, ejection fraction; FS, fractional shortening.

	**STRN$^{+/+}$/AcPBS (n=16)**		**STRN$^{+/-}$/AcPBS (n=10)**		**STRN$^{+/+}$/AngII (n=15)**		**STRN$^{+/-}$/AngII (n=16)**	
**3 d**								
	**Mean**	**SD**	**Mean**	**SD**	**Mean**	**SD**	**Mean**	**SD**
Heart Rate (bpm)	524	39	505	34	534	33	533	36
SV (µl)	41.62	9.01	42.19	6.64	36.18	6.95	38.00	7.92
CO (ml/min)	21.75	5.60	21.27	6.74	19.29	7.44	20.25	7.19
EF (%)	53.28	3.65	56.27	4.58	57.39	4.93	58.53	4.89
FS (%)	27.30	4.67	29.28	3.48	29.88	3.72	30.70	4.43
LVID; d (mm)	3.032	0.192	2.913	0.270	2.675	0.254	2.691	0.339
LVID; s (mm)	4.174	0.246	4.116	0.225	3.816	0.245	3.872	0.339
LVAW; d (mm)	1.052	0.081	1.051	0.112	1.171	0.099	1.103	0.090
LVAW; s (mm)	0.820	0.071	0.805	0.083	0.919	0.070	0.856	0.073
LVPW; d (mm)	0.988	0.085	0.999	0.074	1.193	0.139	1.164	0.123
LVPW; s (mm)	0.698	0.058	0.704	0.041	0.879	0.126	0.825	0.098
WT; d (mm)	2.040	0.146	2.050	0.157	2.364	0.221	2.267	0.202
WT; s (mm)	1.518	0.112	1.509	0.102	1.799	0.181	1.681	0.151
**7 d**	**Mean**	**SD**	**Mean**	**SD**	**Mean**	**SD**	**Mean**	**SD**
Heart Rate (bpm)	523	34	515	33	546	38	529	53
SV (µl)	42.54	7.43	43.27	6.78	39.74	10.93	41.09	7.74
CO (ml/min)	22.24	4.86	22.27	5.17	21.61	6.47	21.84	6.75
EF (%)	57.95	3.29	56.38	3.46	59.12	4.55	61.36	4.87
FS (%)	30.28	4.23	29.33	3.89	31.10	5.70	32.71	5.19
LVID; d (mm)	2.839	0.226	2.941	0.228	2.698	0.343	2.641	0.314
LVID; s (mm)	4.072	0.261	4.157	0.228	3.912	0.413	3.919	0.311
LVAW; d (mm)	1.092	0.068	1.043	0.064	1.210	0.088	1.206	0.100
LVAW; s (mm)	0.826	0.059	0.813	0.133	0.927	0.078	0.898	0.074
LVPW; d (mm)	1.047	0.083	1.052	0.068	1.184	0.095	1.234	0.169
LVPW; s (mm)	0.713	0.075	0.738	0.042	0.837	0.085	0.862	0.158
WT; d (mm)	2.139	0.123	2.095	0.082	2.394	0.171	2.440	0.259
WT; s (mm)	1.539	0.100	1.551	0.143	1.764	0.110	1.761	0.209

Supplementary Table S6. Echocardiography data for STRN3$^{+/-}$ male mice and wild-type (STRN3$^{+/+}$) male littermates treated with acidified PBS (AcPBS) or 0.8 mg/kg/d AngII for 3 or 7 d: M-mode data. Short axis M-mode images were analysed using VevoLab software. LV, left ventricle; ID, internal diameter; AW, anterior wall; PW, posterior wall; d, diastole; s, systole; wall thickness (WT) = AW+ PW; SV, stroke volume; CO, cardiac output; EF, ejection fraction; FS, fractional shortening.

	**STRN3$^{+/+}$/AcPBS (n=16)**		**STRN3$^{+/-}$/AcPBS (n=10)**		**STRN3$^{+/+}$/AngII (n=15)**		**STRN3$^{+/-}$/AngII (n=16)**	
**3 d**								
	**Mean**	**SD**	**Mean**	**SD**	**Mean**	**SD**	**Mean**	**SD**
Heart Rate (bpm)	520	31	508	37	528	39	525	52
SV (µl)	41.52	6.73	41.17	7.08	37.98	7.18	34.55	6.63
CO (ml/min)	54.83	6.73	60.16	5.28	61.65	9.40	56.03	8.25
EF (%)	28.36	4.29	31.77	3.69	33.02	6.70	28.98	5.30
FS (%)	21.48	3.05	20.97	4.38	20.02	3.72	18.10	3.45
LVID; d (mm)	2.973	0.355	2.699	0.210	2.551	0.391	2.703	0.371
LVID; s (mm)	4.140	0.317	3.955	0.231	3.795	0.316	3.795	0.331
LVAW; d (mm)	1.079	0.075	1.098	0.138	1.249	0.163	1.221	0.180
LVAW; s (mm)	0.772	0.065	0.773	0.086	0.890	0.106	0.897	0.138
LVPW; d (mm)	1.034	0.052	1.070	0.109	1.259	0.185	1.220	0.230
LVPW; s (mm)	0.713	0.035	0.733	0.083	0.898	0.127	0.923	0.178
WT; d (mm)	2.113	0.119	2.168	0.232	2.508	0.316	2.441	0.394
WT; s (mm)	1.485	0.086	1.506	0.125	1.788	0.199	1.821	0.294
**7 d**	**Mean**	**SD**	**Mean**	**SD**	**Mean**	**SD**	**Mean**	**SD**
Heart Rate (bpm)	533	42	521	35	539	48	534	34
SV (µl)	42.79	5.38	42.98	7.60	41.75	7.13	39.76	5.10
CO (ml/min)	59.23	6.87	61.24	7.05	61.63	6.35	65.24	3.46
EF (%)	31.25	4.76	32.70	5.28	32.92	4.56	35.25	2.50
FS (%)	22.74	2.84	22.43	4.63	22.50	4.15	21.23	3.24
LVID; d (mm)	2.802	0.373	2.697	0.307	2.650	0.273	2.445	0.209
LVID; s (mm)	4.063	0.311	4.002	0.276	3.944	0.256	3.770	0.236
LVAW; d (mm)	1.130	0.085	1.126	0.119	1.210	0.110	1.294	0.095
LVAW; s (mm)	0.789	0.068	0.781	0.071	0.876	0.089	0.913	0.090
LVPW; d (mm)	1.113	0.084	1.071	0.139	1.258	0.151	1.290	0.164
LVPW; s (mm)	0.741	0.050	0.728	0.058	0.868	0.108	0.929	0.165
WT; d (mm)	2.243	0.160	2.197	0.243	2.468	0.243	2.583	0.243
WT; s (mm)	1.530	0.091	1.509	0.111	1.744	0.163	1.842	0.208

Supplementary Table S7. Echocardiography data for STRN+/- and STRN3+/- male mice and wild-type (STRN+/+ and STRN3+/+) male littermates treated with acidified PBS (AcPBS) or 0.8 mg/kg/d AngII for 7 d: B-mode data and speckle-tracking. Long axis B-mode images were analysed using speckle-tracking software. EDV, End diastolic volume; ESV, End systolic volume; EDLVM, End diastolic left ventricle mass; ESLVM, End systolic left ventricle mass; GLS, global longitudinal strain; GCS, Global circumferential strain; SV, stroke volume; CO, cardiac output; EF, ejection fraction; FS, fractional shortening.

	**STRN+/+/AcPBS (n=16)**		**STRN+/-/AcPBS (n=10)**		**STRN+/+/AngII (n=15)**		**STRN+/-/AngII (n=16)**	
	**Mean**	**SD**	**Mean**	**SD**	**Mean**	**SD**	**Mean**	**SD**
Heart Rate (bpm)	507	35	502	38	541	45	526	49
SV (µl)	29.23	5.17	28.53	5.11	25.68	5.62	27.55	5.01
CO (ml/min)	14.81	2.75	14.34	2.93	13.82	2.95	14.44	2.77
EF (%)	58.93	7.05	58.94	5.63	58.19	6.84	61.99	6.51
FS (%)	31.68	7.28	30.46	5.10	30.96	4.65	33.88	5.27
EDV (µl)	49.58	8.16	48.05	6.21	44.21	9.66	44.80	9.51
ESV (µl)	20.35	5.27	19.52	3.24	18.53	5.82	17.25	5.49
EDLVM (mg)	57.23	5.12	54.27	4.70	69.82	6.81	63.32	7.95
ESLVM (mg)	59.03	5.17	55.47	4.94	72.29	7.61	65.45	9.16
GLS (%)	-19.07	2.66	-19.65	4.71	-17.86	5.06	-19.78	3.70
GCS (%)	-21.45	3.73	-21.47	2.15	-19.61	3.25	-22.01	5.01
	**STRN3+/+ /AcPBS (n=13)**		**STRN3+/- /AcPBS (n=9)**		**STRN3+/+/AngII (n=14)**		**STRN3+/-/AngII (n=10)**	
	**Mean**	**SD**	**Mean**	**SD**	**Mean**	**SD**	**Mean**	**SD**
Heart Rate (bpm)	517	40	520	46	529	51	517	31
SV (µl)	28.78	5.20	28.15	5.31	27.87	4.67	25.41	5.72
CO (ml/min)	14.85	2.72	14.80	4.11	14.69	2.59	13.08	2.75
EF (%)	58.04	6.77	61.92	3.13	58.94	7.01	62.44	6.16
FS (%)	31.61	7.49	34.72	4.31	31.84	7.56	33.24	7.33
EDV (µl)	50.60	13.75	45.20	7.87	47.45	8.97	40.79	9.88
ESV (µl)	21.82	9.25	17.05	3.08	19.57	6.22	15.38	5.34
EDLVM (mg)	53.99	6.23	53.83	5.42	68.06	7.48	69.43	7.30
ESLVM (mg)	57.12	7.14	56.01	5.81	71.12	7.46	73.15	7.28
GLS (%)	-19.46	1.96	-20.56	2.92	-18.79	4.11	-22.72	4.00
GCS (%)	-20.39	3.51	-23.53	3.81	-22.95	2.46	-22.80	3.68

Supplementary Table S8. Echocardiography data for $STRN3^{fl/fl}/Cre^{+/-}$ mice: M-mode data. Male mice (8 wks) were treated with corn-oil vehicle (COV) or 40 mg/kg tamoxifen (Tx) 4 days amd then with acidified PBS (AcPBS) or 0.8 mg/kg/d AngII in AcPBS. Short axis M-mode images were analysed using VevoLab software. LV, left ventricle; ID, internal diameter; AW, anterior wall; PW, posterior wall; d, diastole; s, systole; wall thickness (WT) = AW+ PW; SV, stroke volume; CO, cardiac output; EF, ejection fraction; FS, fractional shortening.

	COV/AcPBS		Tx/AcPBS		COV/AngII		Tx/AngII	
**Baseline**	**Mean**	**SD**	**Mean**	**SD**	**Mean**	**SD**	**Mean**	**SD**
Heart Rate (bpm)	481	27	479	30	485	22	492	26
SV (µl)	44.48	6.53	44.44	6.18	45.37	6.23	41.20	6.24
CO (ml/min)	57.28	6.55	57.40	4.50	60.48	5.30	59.01	5.51
EF (%)	30.04	4.54	29.99	2.94	32.16	3.72	31.11	3.64
FS (%)	21.44	3.72	21.31	3.68	21.94	3.02	20.15	2.38
LVID; d (mm)	2.929	0.279	2.932	0.328	2.793	0.164	2.767	0.325
LVID; s (mm)	4.180	0.241	4.177	0.330	4.115	0.154	4.003	0.313
LVAW; d (mm)	0.949	0.055	0.974	0.038	0.990	0.056	0.978	0.047
LVAW; s (mm)	0.693	0.028	0.696	0.043	0.720	0.031	0.722	0.028
LVPW; d (mm)	0.954	0.072	0.930	0.060	0.959	0.049	0.961	0.049
LVPW; s (mm)	0.637	0.063	0.642	0.041	0.644	0.025	0.653	0.039
WT; d (mm)	1.904	0.107	1.904	0.087	1.949	0.097	1.939	0.091
WT; s (mm)	1.329	0.083	1.338	0.072	1.364	0.048	1.374	0.058
**3 d**	**Mean**	**SD**	**Mean**	**SD**	**Mean**	**SD**	**Mean**	**SD**
Heart Rate (bpm)	502	28	496	29	506	21	505	45
SV (µl)	46.38	8.99	47.36	7.12	39.34	5.58	40.99	8.15
CO (ml/min)	59.78	8.29	60.04	4.78	66.91	6.04	67.97	10.76
EF (%)	31.79	5.74	31.82	3.50	36.63	4.38	37.92	8.11
FS (%)	23.25	4.67	23.61	4.52	19.92	2.93	20.59	3.89
LVID; d (mm)	2.855	0.397	2.862	0.215	2.365	0.283	2.357	0.491
LVID; s (mm)	4.176	0.354	4.197	0.232	3.719	0.253	3.768	0.378
LVAW; d (mm)	1.033	0.080	1.043	0.058	1.180	0.112	1.138	0.119
LVAW; s (mm)	0.720	0.050	0.744	0.043	0.873	0.065	0.803	0.053
LVPW; d (mm)	0.993	0.067	1.005	0.076	1.210	0.115	1.139	0.123
LVPW; s (mm)	0.667	0.059	0.684	0.059	0.818	0.122	0.738	0.058
WT; d (mm)	2.026	0.132	2.048	0.112	2.390	0.214	2.277	0.233
WT; s (mm)	1.387	0.092	1.429	0.096	1.691	0.185	1.541	0.091
**7 d**	**Mean**	**SD**	**Mean**	**SD**	**Mean**	**SD**	**Mean**	**SD**
Heart Rate (bpm)	531	52	506	36	508	54	527	45
SV (µl)	44.56	7.98	45.64	5.26	38.49	10.59	37.49	6.47
CO (ml/min)	57.67	8.51	59.35	5.18	61.65	8.59	60.91	9.30
EF (%)	30.33	5.69	31.34	3.65	33.00	6.04	32.47	6.72
FS (%)	23.87	5.79	23.21	3.91	19.33	5.00	19.63	2.90
LVID; d (mm)	2.900	0.249	2.860	0.235	2.562	0.408	2.586	0.469
LVID; s (mm)	4.164	0.148	4.161	0.199	3.801	0.428	3.804	0.390
LVAW; d (mm)	1.062	0.090	1.049	0.069	1.159	0.093	1.035	0.074
LVAW; s (mm)	0.764	0.058	0.747	0.043	0.851	0.078	0.755	0.049
LVPW; d (mm)	1.018	0.117	1.013	0.089	1.177	0.159	1.034	0.122
LVPW; s (mm)	0.697	0.083	0.693	0.061	0.873	0.151	0.684	0.040
WT; d (mm)	2.079	0.185	2.062	0.131	2.337	0.240	2.070	0.168
WT; s (mm)	1.461	0.131	1.440	0.099	1.723	0.222	1.439	0.085

Supplementary Table S9. Echocardiography data for STRN3$^{fl/fl}$/Cre$^{+/-}$ mice: B-mode data and speckle-tracking. Male mice (8 wks) were treated with corn-oil vehicle (COV) or 40 mg/kg tamoxifen (Tx) 4 days before minipumps were implanted for delivery of acidified PBS (AcPBS) or 0.8 mg/kg/d AngII in AcPBS for 7 d. Long axis B-mode images were analysed using speckle-tracking software. Tx, tamoxifen; EDV, End diastolic volume; ESV, End systolic volume; EDLVM, End diastolic left ventricle mass; ESLVM, End systolic left ventricle mass; GLS, global longitudinal strain; GCS, Global circumferential strain; SV, stroke volume; CO, cardiac output; EF, ejection fraction; FS, fractional shortening.

	**COV/AcPBS**		**Tx/AcPBS**		**COV/AngII**		**Tx/AngII**	
	Mean	SD	Mean	SD	Mean	SD	Mean	SD
**Baseline**								
Heart Rate (bpm)	460	27	465	25	468	32	476	26
SV (µl)	33.26	5.25	34.35	4.56	32.10	2.68	32.60	4.42
CO (ml/min)	15.36	2.82	15.96	2.23	15.04	1.89	15.43	1.67
EF (%)	56.56	6.96	56.64	3.44	57.21	4.71	58.42	5.15
FS (%)	30.20	6.91	30.39	3.74	31.37	4.54	31.26	4.14
EDV (µl)	58.76	6.92	60.69	8.28	56.20	4.25	56.26	9.59
ESV (µl)	25.50	5.09	26.34	4.57	24.11	3.79	23.65	6.47
EDLVM (mg)	54.62	3.78	53.42	3.39	52.82	2.94	52.05	3.08
ESLVM (mg)	57.36	3.85	56.82	4.77	54.63	3.53	54.95	3.08
GLS (%)	-19.96	2.84	-20.84	2.71	-19.27	2.64	-20.92	3.20
GCS (%)	-21.19	3.27	-21.68	2.51	-21.65	2.71	-21.15	2.96
**7 d**								
Heart Rate (bpm)	514	56	496	38	490	59	515	62
SV (µl)	33.40	6.21	33.09	4.79	27.74	7.67	28.14	5.35
CO (ml/min)	17.26	3.90	16.34	2.16	13.45	3.74	14.30	2.12
EF (%)	60.64	8.77	59.54	5.69	56.44	11.08	59.95	6.41
FS (%)	33.88	5.48	31.30	5.47	31.45	8.26	36.14	6.01
EDV (µl)	54.94	8.31	55.63	9.49	50.47	13.99	47.69	12.79
ESV (µl)	21.54	6.55	22.54	6.21	22.73	8.55	19.55	7.94
EDLVM (mg)	55.58	4.65	54.17	4.56	71.19	15.02	58.98	4.37
ESLVM (mg)	58.04	5.17	57.22	4.79	72.26	15.07	60.15	4.38
GLS (%)	-20.57	6.10	-20.37	3.09	-16.90	3.76	-16.74	3.10
GCS (%)	-23.04	4.23	-22.75	3.28	-22.03	3.48	-21.28	5.52

Supplementary Figure S1. Global heterozygous STRN3 knockout in mice. A, "Knockout-first" strategy for global deletion of STRN3 in mice involved positioning of a STOP cassette flanked by FRT sites upstream of a critical exon that was also flanked with LoxP sites. **B,** Experimental approach for assessment of effects of STRN3 deletion on cardiac function. Homozygous global knockout of STRN3 is embryonic lethal, so heterozygote STRN3$^{+/-}$ male mice (8 wks) were used in comparison with wild-type (WT) littermates from each colony. Following baseline echocardiography (echo), minipumps were implanted for delivery of acidified PBS vehicle (AcPBS) or 0.8 mg/kg/d angiotensin II (AngII). Following echocardiography at 7 d, mice were sacrificed. **C-D,** Heart powders were used for immunoblotting (40 µg protein per lane). Representative immunoblots of the striatin isoforms and GAPDH (**C**) are shown with densitometric analysis (**D**). Results are relative to GAPDH and normalised to the means for WT mice treated with AcPBS. Individual datapoints are plotted with means ± SEM. N.B. The upper band of the STRN blot used for densitometry correlates with the predicted molecular weight of STRN protein (110k Da).

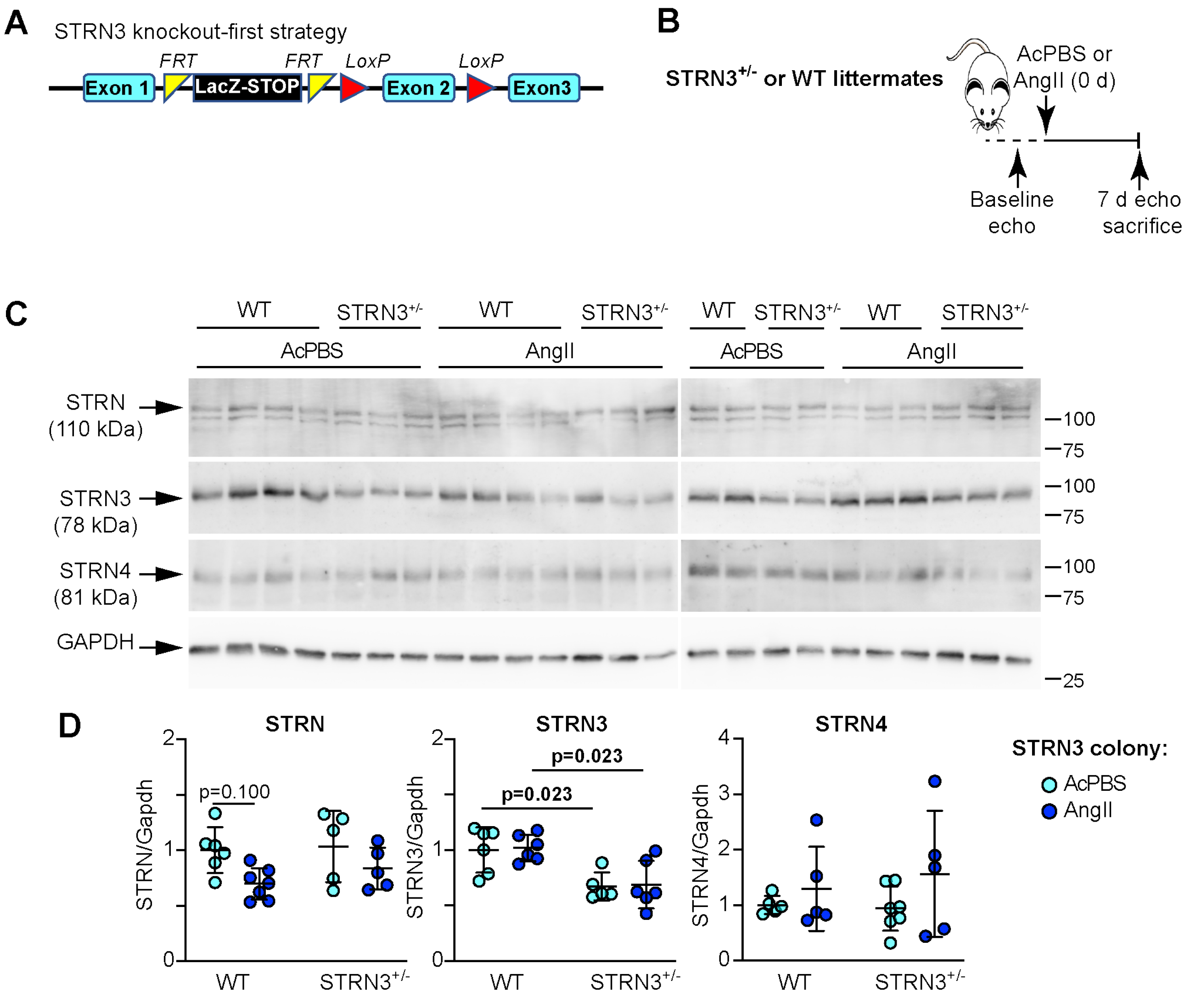

Supplementary Figure S2. Heterozygous global deletion of STRN does not affect the hypertrophic response to AngII. Male mice (8 wks) heterozygote for STRN3 knockout ($STRN3^{+/}$) and wild-type (WT) littermates from each colony were treated with acidified PBS (AcPBS) vehicle or AngII (0.8 mg/kg/d). Cardiac function and dimensions were assessed by echocardiography using M-mode imaging of the short axis at 3 d (**A**) or 7 d (**B**), or B-mode imaging of the long axis at 7 d with speckle-tracking and strain analysis (C). For M-mode imaging, diastolic values for left ventricle (LV) wall thickness (WT) or internal diameter (ID) are shown and end diastolic LV mass and volume are provided for B-mode imaging. Cardiac function measurements are shown for both M-mode and B-mode for comparison. Individual datapoints are plotted with means ± SEM. Statistical analysis used 2-way ANOVA with Holm-Sidak's post-test. (N.B. All echocardiography data are provided in **Supplementary Tables S4, S6 and S7**).

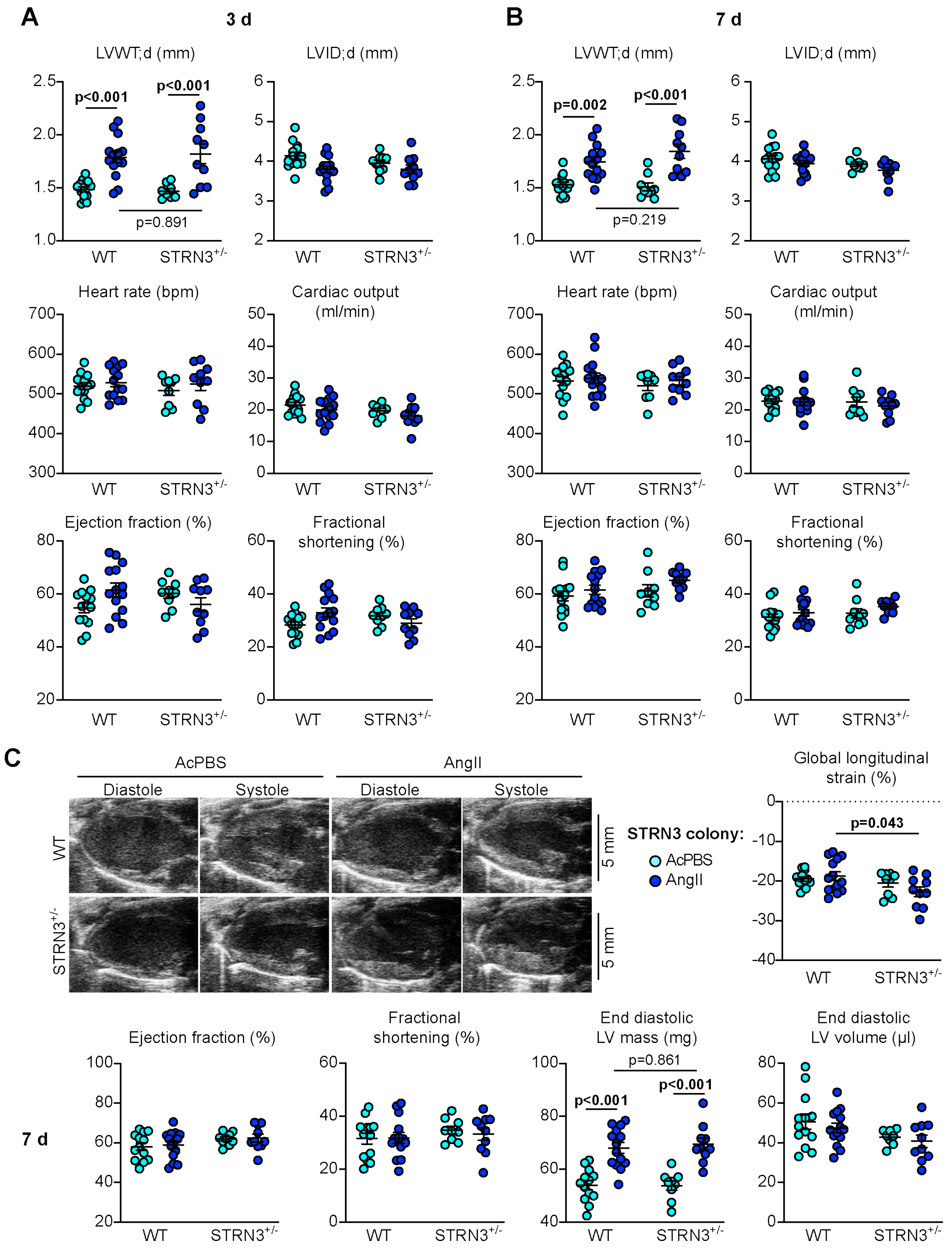
A
3 d
B
7 d
LVWT;d (mm)
LVID;d (mm)
p<0.001
p<0.001
p=0.891
p=0.002
p<0.001
p=0.219
WT
STRN3+/-
Heart rate (bpm)
Cardiac output (ml/min)
Ejection fraction (%)
Fractional shortening (%)
C
AcPBS
AngII
Diastole
Systole
WT
STRN3+/-
5 mm
STRN3 colony:
AcPBS
AngII
Global longitudinal strain (%)
p=0.043
7 d
End diastolic LV mass (mg)
p=0.861
p<0.001
p<0.001
End diastolic LV volume (µl)

Supplementary Figure S3. Heterozygous global deletion of STRN3 does not affect cardiomyocyte hypertrophy or cardiac fibrosis induced by AngII. 8 wk male $STRN^{+/-}$ mice, plus their respective wild-type (WT) littermates were treated with acidified PBS (AcPBS) vehicle or 0.8 mg/kg/d AngII (7 d). **A-B,** Hearts were fixed and sections stained with haemotoxylin and eosin. Representative images (**A**) show areas from the outer perimeter of the left ventricular wall opposite the interventricular septum and cardiomyocyte cross-sectional areas are shown (**B**). **C,** RNA was extracted from mouse heart powders and analysed by qPCR for *Myh7* and *Nppb* mRNAs. **D-E,** Hearts were fixed and sections stained with picrosirius red. Representative short axis views of the whole heart are shown for wild-type and $STRN^{+/-}$ mice treated with AcPBS or AngII (**D**). For AngII-treated hearts, the minimum or maximum response is shown in the middle and lower panels, respectively. **E,** Interstitial fibrosis was measured using Image J and is presented as the % of the total area (excluding regions around the blood vessels). Perivascular fibrosis was scored (1: negligible increase in fibrosis around any vessel; 2: mild to moderate fibrosis around 1 or more vessels; 3: Significant fibrosis permeating tissue around 1 or more vessels; 4: extensive fibrosis around multiple vessels, penetrating into the myocardium). A scoring system was used for the latter because of the variation in numbers of vessels seen in different heart sections. **F,** RNA was extracted from mouse heart powders and analysed by qPCR for fibrosis mRNAs as indicated. Results for qPCR are relative to GAPDH and normalised to the means for WT mice treated with AcPBS. Individual datapoints are plotted with means ± SD. Statistical analysis used 2-way ANOVA with Holm-Sidak's post-test.

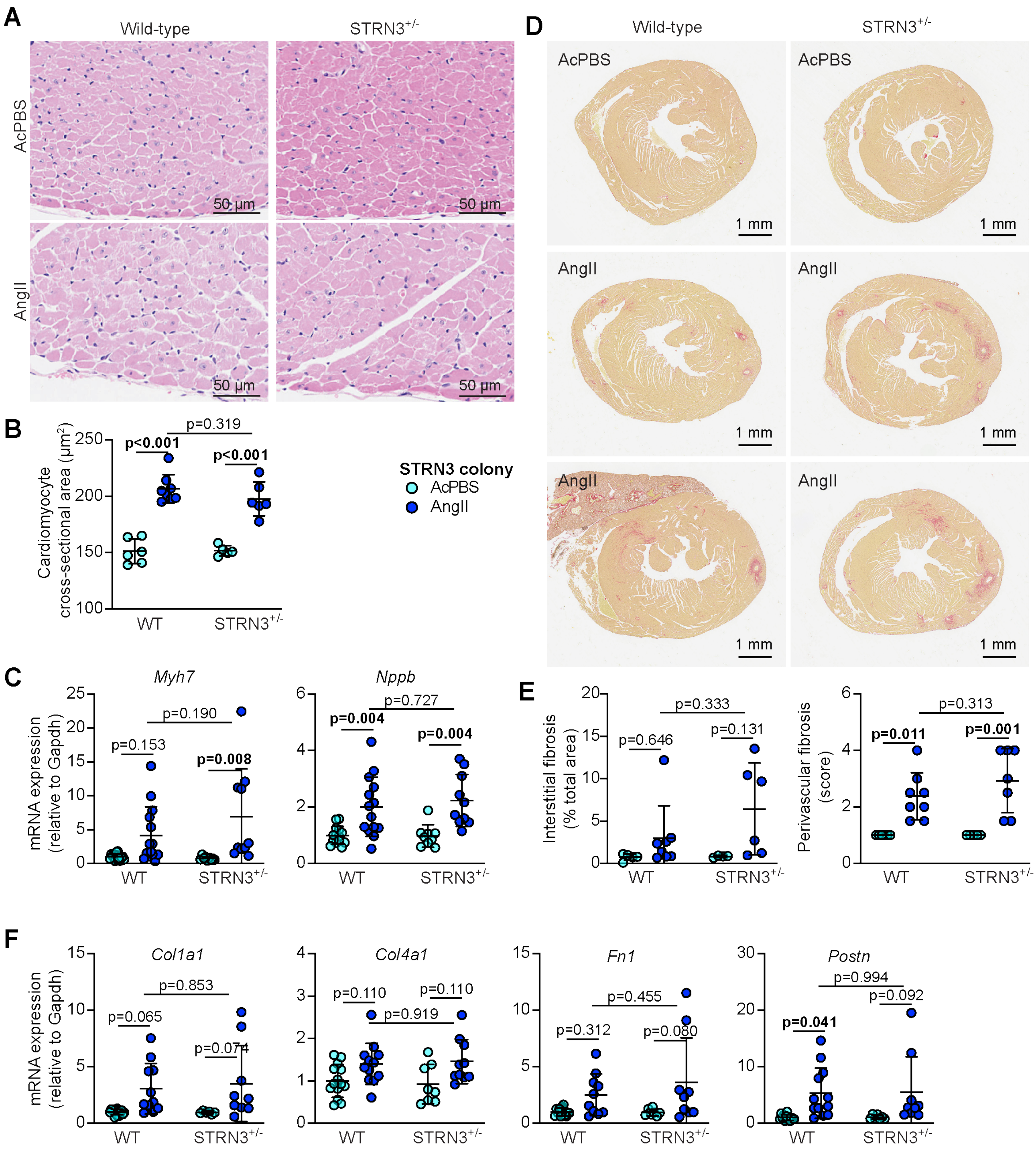

A
Wild-type
STRN3+/-
AcPBS
AngII
50 μm
B
Cardiomyocyte cross-sectional area (μm²)
p=0.319
p<0.001
p<0.001
WT
STRN3+/-
STRN3 colony
AcPBS
AngII
C
Myh7
Nppb
mRNA expression (relative to Gapdh)
p=0.190
p=0.153
p=0.008
p=0.727
p=0.004
p=0.004
D
Wild-type
STRN3+/-
AcPBS
AngII
1 mm
E
Interstitial fibrosis (% total area)
p=0.333
p=0.646
p=0.131
Perivascular fibrosis (score)
p=0.313
p=0.011
p=0.001
F
Col1a1
Col4a1
Fn1
Postn
p=0.853
p=0.065
p=0.074
p=0.110
p=0.110
p=0.919
p=0.455
p=0.312
p=0.080
p=0.994
p=0.092
p=0.041

Supplementary Figure S4. Full images for immunoblots in Figure 1C. Proteins were separated on 10% polyacrylamide gels. Red boxes highlight the bands of interest.

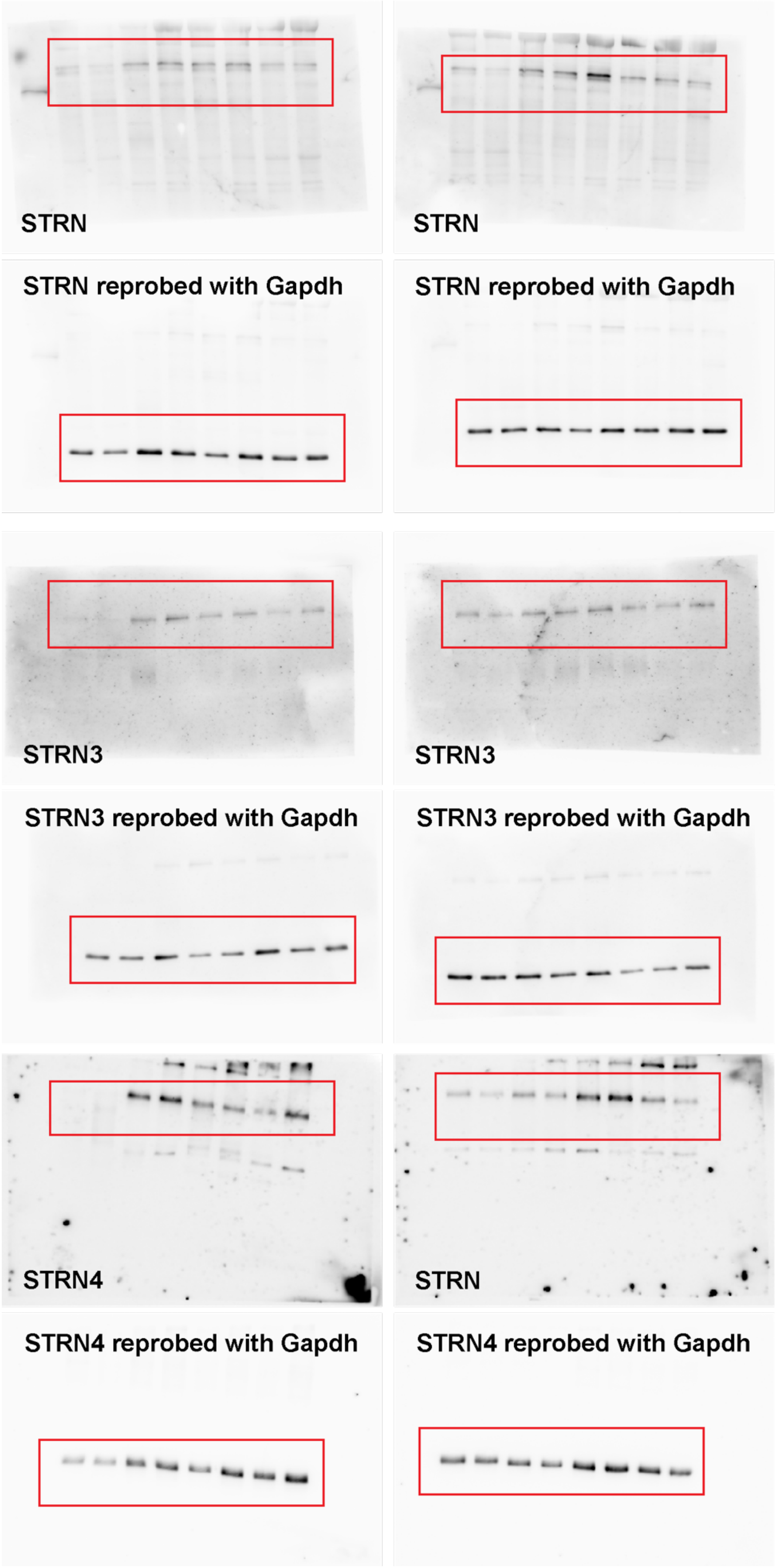

Supplementary Figure S5. Full images for immunoblots in Figure 1G. Proteins were separated on 8% and 12% polyacrylamide gels for striatins and GAPDH, respectively. Red boxes highlight the bands of interest.

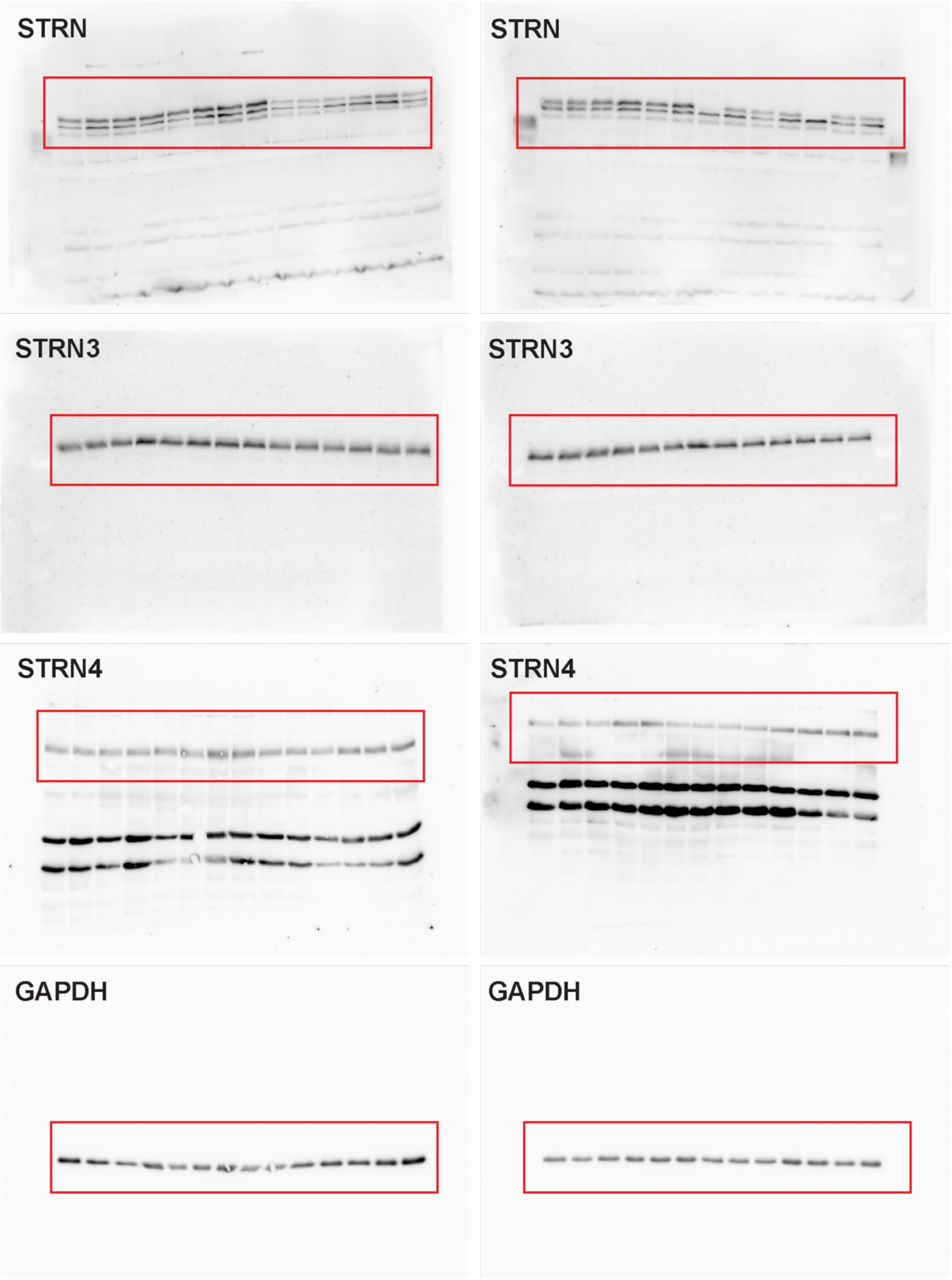

Supplementary Figure S6. Full images for immunoblots in Supplementary Figure S1C. Proteins were separated on 8% and 12% polyacrylamide gels for striatins and GAPDH, respectively. Red boxes highlight the bands of interest.

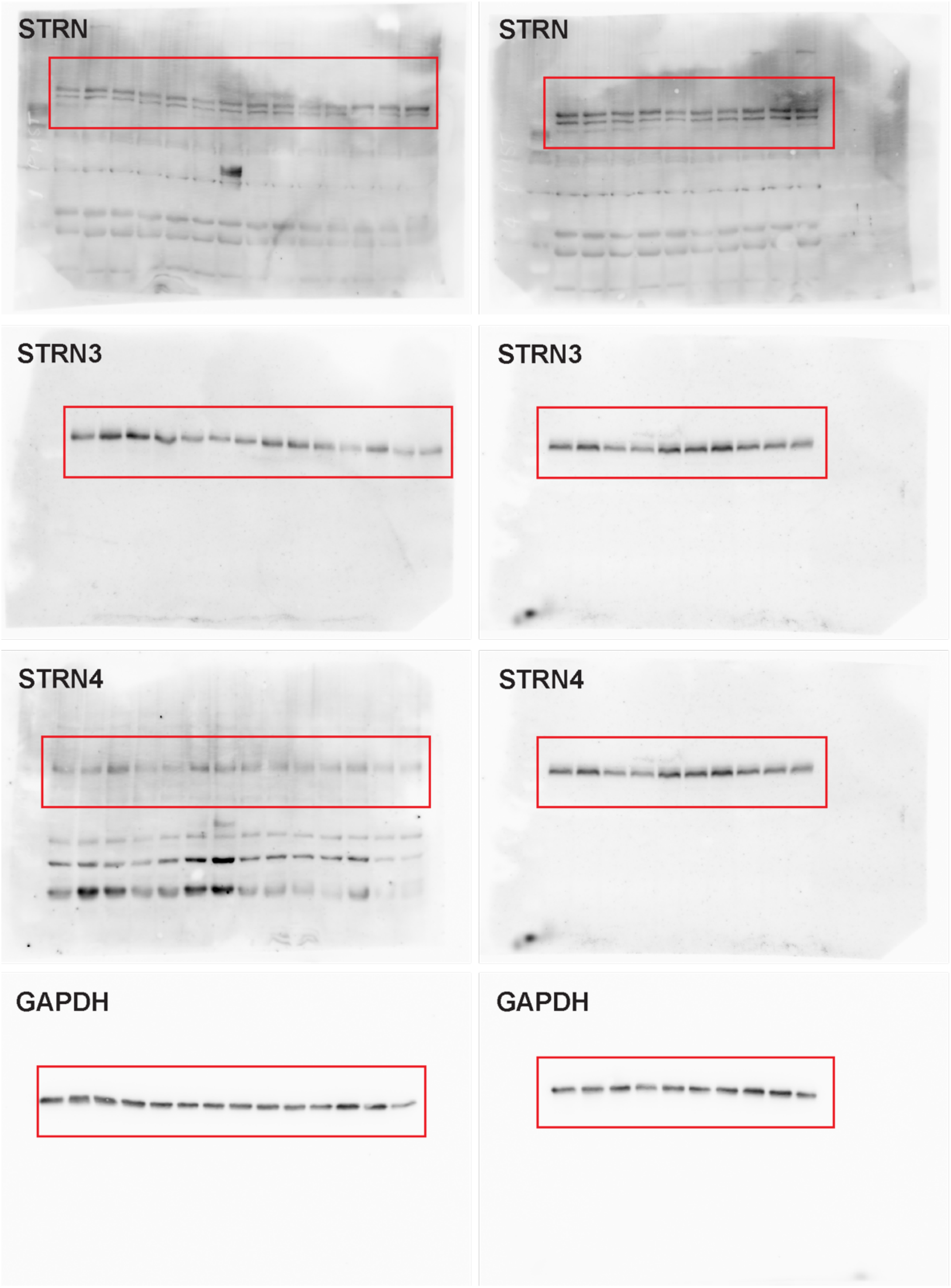

Supplementary Figure S7. Full images for immunoblots in Figure 5D. Proteins were separated on 8% and 10% polyacrylamide gels for striatins and GAPDH, respectively. (N.B. Two half blots for GAPDH from different experiments were imaged together giving upper and lower bands). Red boxes highlight the bands of interest.

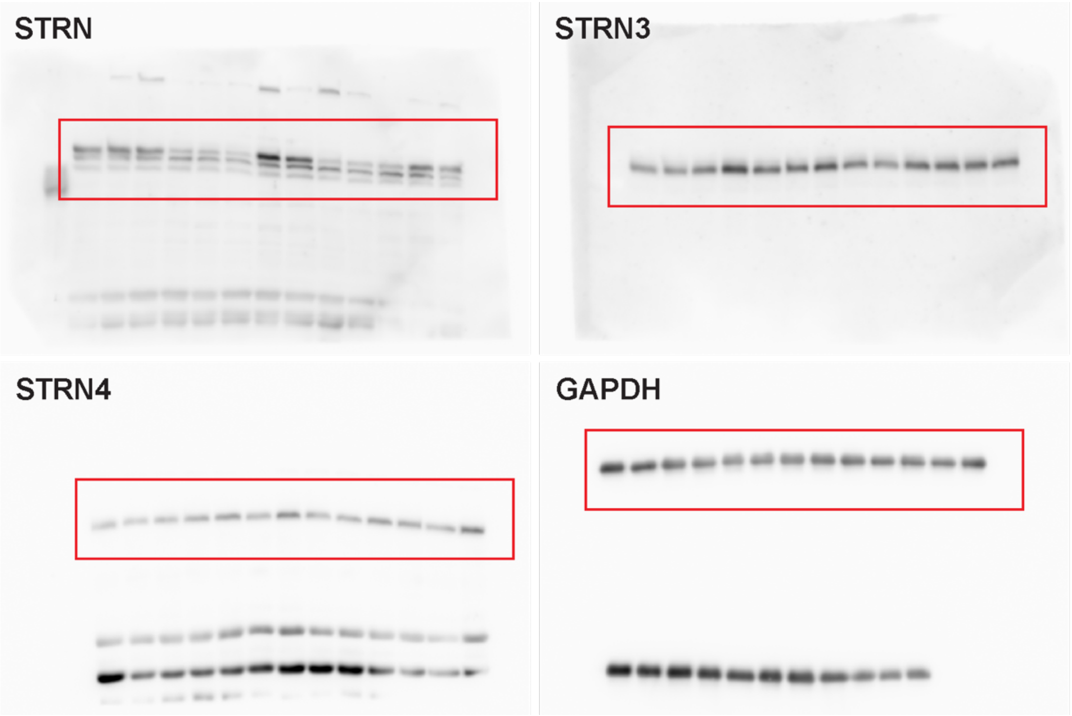

Supplement: Supplementary Figures S1-S7 and Tables S1-S9 [file CS-2024-0496_supp.pdf]
